# Sensor-Based Cross-Modal Spatiotemporal Alignment and Causal Profit Modeling for Agricultural Input Optimization

**DOI:** 10.3390/s26154994

**Published:** 2026-08-06

**Authors:** Zhengjie Fu, Yuheng Zhang, Shuangze Yu, Wen Lv, Shihan Ru, Wenbo Wang, Yihong Song

**Affiliations:** 1 China Agricultural University, Beijing 100083, China; 2 Peking University, Beijing 100871, China

**Keywords:** smart agriculture, multimodal agricultural sensing, agricultural economic returns, precision irrigation and fertilization, reinforcement learning decision-making

## Abstract

As smart agriculture gradually shifts from single-yield monitoring toward the coordinated optimization of input efficiency, resource conservation, and farm profitability, the use of multi-source agricultural data to support precise water, fertilizer, and pesticide inputs has become an important issue. To address the inconsistent sampling frequencies, heterogeneous semantic scales, and difficulty in directly modeling input–profit relationships among field images, meteorological environments, soil states, and agricultural management records, a Multimodal Agricultural Input–Output Optimization Network, termed MAION, is proposed. In this framework, a unified plot–time-window agricultural state representation is constructed through a cross-modal spatiotemporal alignment module. The potential effects of different input behaviors on yield, cost, and net profit are estimated through an input–output causal profit modeling module, and profit-driven reinforcement learning is further used to generate input strategies oriented toward long-term net profit maximization. Since the task belongs to yield, cost, and profit regression prediction and continuous agricultural decision optimization rather than classification or recognition, classification metrics such as accuracy, precision, and recall were not adopted. Instead, RMSE, MAE, $R^{2}$, Net Profit Improvement, Input–Output Ratio, Cumulative Reward, Policy Stability, and Regret were used for evaluation. Experimental results show that MAION achieves the best performance in yield, cost, and net profit prediction, with RMSE values of 0.587, 0.531, and 0.648, respectively, and corresponding $R^{2}$ values of 0.914, 0.891, and 0.883. These results are markedly superior to those of Random Forest, XGBoost, LSTM, GRU, Transformer, Multimodal Transformer, and reinforcement learning baseline models. In the economic decision-making experiment, MAION achieves a Net Profit Improvement of 17.68%, an Input–Output Ratio of 1.71, a Cost Efficiency Gain of 15.46%, and a Cumulative Reward of 301.27, while obtaining the lowest policy fluctuation and regret. The results indicate that the proposed framework can provide effective data-driven decision support for precision input, cost control, and profit optimization in smart agriculture.

## 1. Introduction

With the continuous advancement of smart agriculture, agricultural production is shifting from experience-dependent judgment toward integrated management supported by data, models, and intelligent decision-making [1]. Internet of Things sensors, unmanned aerial vehicle remote sensing, field image acquisition devices, meteorological monitoring systems, and agricultural management platforms have been increasingly deployed in farmland and orchard production scenarios, enabling continuous recording of soil moisture, temperature and humidity, light intensity, rainfall, crop growth status, pest and disease conditions, irrigation and fertilization records, and pesticide application information [2]. These data provide a fine-grained perception basis for precision agriculture and allow crop growth processes and farmland environmental changes to be understood more comprehensively [3]. However, data availability does not necessarily mean that collected data can be directly transformed into effective decisions [4]. In practical production, managers are usually concerned not only with the accurate prediction of a single indicator, but also with how fertilization, irrigation, and pesticide application should be arranged under current crop status, meteorological conditions, and soil background so that input costs can be controlled while yield and quality are maintained [5]. With increasing production costs, tightening resource constraints, and intensifying climate variability, simply pursuing yield maximization is no longer sufficient for modern agricultural management. A more reasonable balance among yield, cost, resource utilization, and net profit has therefore become an important issue in smart agricultural decision-making research [6].

Traditional agricultural management usually relies on farmer experience, expert rules, and statistical analysis models [7]. Experience-driven methods are widely used because of their direct operation and low implementation cost, but their effectiveness depends heavily on personal experience and shows limited adaptability to climate variation, plot heterogeneity, and crop growth-stage changes [8]. Expert systems can provide recommendations for irrigation, fertilization, and pest or disease control according to predefined rules; however, their rule structures are usually fixed and may lack flexibility when complex coupling relationships exist among environmental variables, crop states, and input behaviors. Statistical models and production function models can analyze relationships between agricultural inputs and outputs, but they often rely on linear or static assumptions and are insufficient for describing nonlinear responses, time-lag effects, and stage-dependent differences in agricultural systems [9]. Moreover, many traditional methods still focus on a single production indicator, such as yield, disease incidence, or soil moisture, while the joint relationships among crop status, environmental conditions, input behavior, and economic return are rarely considered. Therefore, traditional methods remain inadequate for multi-source data fusion, dynamic input optimization, and profit-oriented intelligent decision-making [10].

Deep learning has provided a new technical pathway for smart agriculture and has been widely applied to crop recognition, pest and disease detection, yield prediction, quality assessment, and environmental monitoring [11]. Convolutional neural networks can extract crop growth, canopy structure, and disease features from field images and unmanned aerial vehicle remote sensing images, while recurrent neural networks, long short-term memory networks, and Transformer models can process meteorological, soil, and crop growth time-series data. Multimodal learning methods have further attempted to integrate images, sensor data, and management records to improve agricultural state perception and yield prediction [12]. These studies have promoted the transition from manual observation to automated analysis, but limitations remain in input optimization. Several recent studies have explored causal modeling, reinforcement learning, and multimodal learning for agricultural or related complex decision-making tasks. Zhang et al. [13] proposed the CMRL causal meta-reinforcement learning framework, integrating reinforcement learning feedback, causal intervention, and meta-learning optimization for multimodal remote sensing classification, with overall accuracies of 86.27%, 95.51%, and 99.41% on the MUUFL, Houston, and Trento datasets, respectively. Liu et al. [14] proposed the ASGO-PAPIS framework, which combined deep learning and causal inference for multidimensional dairy production cost analysis and improved Precision, NDCG, and MAP by up to approximately 5 percentage points. Zhang et al. [15] developed a cross-modal attention and PPO reinforcement learning framework for pesticide application optimization by fusing unmanned aerial vehicle hyperspectral data, soil meteorological data, and pest monitoring data, achieving an RMSE of 0.162, a spraying accuracy of 88.3%, an 18.3% reduction in pesticide usage, and a pest suppression effect of 77.9%.

Although multimodal learning, causal inference, and reinforcement learning have each demonstrated considerable potential for intelligent agricultural management, they address different stages of the decision-making process and therefore exhibit complementary strengths as well as inherent limitations. Multimodal fusion effectively integrates heterogeneous information from crop images, environmental observations, soil conditions, and management records, enabling comprehensive agricultural state perception [16]. However, it primarily models statistical correlations and cannot reliably determine how changes in irrigation, fertilization, or pesticide application causally affect agricultural productivity and economic returns. Causal inference alleviates confounding effects caused by weather variability, soil heterogeneity, and management differences, providing more reliable estimates of intervention effects. Nevertheless, existing causal approaches mainly focus on effect estimation rather than generating optimal sequential management strategies throughout an entire growing season [17]. Reinforcement learning is naturally suitable for optimizing long-term agricultural decisions, yet its performance strongly depends on accurate state representations and reliable reward signals. Inaccurate perception or biased intervention estimation may consequently result in unstable or economically suboptimal policies. Therefore, these three paradigms should be regarded as complementary rather than competing approaches. A unified framework integrating multimodal perception, causal reasoning, and reinforcement learning can simultaneously achieve comprehensive agricultural state representation, reliable intervention effect estimation, and long-term profit-oriented decision optimization.

Accordingly, a multimodal causal reinforcement learning framework for agricultural input–profit optimization, namely the Multimodal Agricultural Input–Output Optimization Network, abbreviated as MAION, is proposed in this study. Visual data, environmental data, soil data, and agricultural management records are used as inputs, while yield, cost, net profit, and optimal input strategies are taken as outputs. An end-to-end modeling method from multi-source agricultural perception to economically optimal decision-making is thereby constructed. Unlike models that focus only on yield prediction or state recognition, MAION incorporates agricultural input behavior, production cost, and economic return into a unified learning objective. Through cross-modal spatiotemporal alignment, causal input–profit modeling, and profit-driven reinforcement learning decision-making, agricultural intelligent perception is connected with practical production decisions, thereby providing an executable modeling strategy for precision input, cost control, and net profit improvement. The main contributions of this study are summarized as follows:

1. To address the lack of unified agricultural state representation caused by heterogeneous data sources with inconsistent temporal and semantic characteristics, a unified multimodal agricultural input–output modeling framework is developed to support profit-oriented decision making.
2. To address the spatiotemporal inconsistency among crop images, environmental observations, soil measurements, and management records, a cross-modal spatiotemporal alignment strategy is introduced to establish consistent multimodal agricultural representations.
3. To address the inability of existing predictive models to distinguish causal intervention effects from statistical correlations, a causal-aware input–profit modeling mechanism is developed to provide more reliable estimation of the economic effects of agricultural management actions.
4. To address the limitation of conventional optimization methods in achieving long-term economic management, a profit-driven reinforcement learning strategy is designed to optimize sequential agricultural input decisions under long-term profit objectives.
5. To address the challenge of robust deployment in practical agricultural environments, comprehensive cross-plot, cross-year, and missing-modality experiments are conducted to validate the generalization capability and practical effectiveness of the proposed framework.

## 2. Related Work

### 2.1. Multimodal Perception Methods in Smart Agriculture

Multimodal perception in smart agriculture refers to the continuous observation and integrated representation of agricultural production systems using heterogeneous data sources, including images, sensors, remote sensing, and management records [18]. Although these data differ in acquisition frequency, spatial coverage, and semantic level, they jointly provide important information for agricultural state perception [19]. Therefore, multimodal perception is valuable not only because more data types are introduced, but also because complementary information from different sources can be used to identify crop growth status, environmental changes, and production risks more reliably [20]. Image-based agricultural perception methods have been widely studied [21]. Convolutional neural networks can automatically extract texture, color, edge, and morphological features from crop images, and have been applied to disease recognition, fruit detection, weed identification, and quality grading [22]. Vision Transformers further improve the representation of long-range regional dependencies and complex background information, thereby enhancing adaptability under occlusion, illumination variation, and scale differences in field environments [23]. Sensor-based time-series data are also important for greenhouse control, soil moisture monitoring, and crop growth trend analysis, where continuous environmental variables need to be modeled dynamically [24]. Long short term memory (LSTM), gated recurrent unit (GRU), and Transformer models have been adopted to capture long-term dependencies among meteorological variables, soil variables, and growth indicators [25]. Remote sensing methods are more commonly used for regional growth estimation, drought monitoring, and yield assessment through spectral features, vegetation indices, and spatial textures [26]. Despite these advances, most existing studies remain centered on a single modality or a single task [27]. Image models usually focus on disease categories, fruit numbers, or phenotypic traits; sensor models are mainly used for environmental trend prediction or soil state estimation; and remote sensing models emphasize regional crop growth variation [28]. Such fragmented strategies are effective for specific tasks, but they are insufficient for describing the coupled relationships among crops, environments, soils, and management behaviors [29]. For agricultural input–profit optimization, crop growth status or environmental risk alone is not enough; it is also necessary to understand how these states influence fertilization, irrigation, and pesticide application, and how such behaviors are ultimately reflected in yield, cost, and net profit [30]. Accordingly, limitations remain in cross-modal spatiotemporal alignment, unified state representation, and feature fusion oriented toward economic decision-making [31,32].

### 2.2. Agricultural Yield Prediction and Input Modeling Methods

Agricultural yield prediction and input modeling aim to estimate crop production by modeling the relationships among environmental conditions, soil properties, management practices, and historical crop growth states [33]. Early studies mainly relied on statistical regression, crop growth models, and production function models to quantify the effects of climate, soil, irrigation, and fertilization on yield [34,35]. Although these methods are interpretable, they have limited ability to capture the nonlinear and dynamic interactions among meteorological conditions, soil characteristics, crop varieties, pests, and management practices that jointly determine agricultural production [36]. Recent studies have introduced machine learning and deep learning methods to improve yield modeling [37]. Tree-based models such as Random Forest and XGBoost, together with support vector machines, have demonstrated robust performance on structured agricultural data [38], while sequential models including LSTM, GRU, and Transformer further improve the modeling of temporal dependencies and long-term environmental effects from multi-source observations [39,40]. These advances have substantially improved prediction accuracy for agricultural production. However, existing studies remain primarily prediction-oriented. Most methods optimize yield without explicitly considering production costs or economic returns, despite the fact that maximizing yield does not necessarily maximize profit [41,42]. In addition, current models generally learn statistical correlations between agricultural inputs and outputs rather than identifying the underlying causal relationships, making them vulnerable to confounding factors such as weather, soil conditions, and management differences [43]. Consequently, they provide limited support for reliable input optimization [44]. To address these limitations, the proposed causal-aware input–profit modeling module treats fertilization, irrigation, and pesticide application as intervenable variables, jointly estimating their effects on yield, cost, and profit from multimodal agricultural state representations, thereby extending agricultural modeling from prediction toward interpretable decision-oriented optimization [45].

### 2.3. Agricultural Economic Optimization and Intelligent Decision-Making Methods

Agricultural economic optimization aims to allocate land, water, fertilizer, pesticides, labor, and other production resources to balance yield, quality, cost, and profit under resource constraints [46]. Traditional approaches mainly rely on production functions, cost–benefit analysis, and input–output efficiency evaluation to quantify the economic effects of agricultural inputs and support production planning [47,48]. Although these methods provide clear economic interpretations, they generally assume relatively stable environmental conditions and fixed input–output relationships, making them difficult to apply to real agricultural systems where weather, market conditions, and crop responses vary continuously across growth stages [49]. Consequently, agricultural input optimization is increasingly regarded as a dynamic sequential decision-making problem rather than a static optimization task [50]. Reinforcement learning (RL) provides an effective framework for learning long-term agricultural management strategies by optimizing cumulative rewards through continuous interactions with the environment [51,52]. Existing studies formulate crop status, soil conditions, meteorological observations, and historical management records as states [53], while irrigation, fertilization, and pesticide application are treated as actions, enabling optimization of long-term objectives such as yield, quality, resource consumption, and economic return [54]. Compared with prediction-based approaches, RL is better suited to stage-dependent agricultural management involving sequential input decisions [55]. Nevertheless, RL alone remains insufficient for agricultural input optimization [56]. Effective policy learning depends on accurate multimodal state representation, while the estimated effects of agricultural inputs are easily confounded by environmental factors such as weather, soil conditions, and field heterogeneity [57]. Therefore, this study integrates multimodal perception, causal profit modeling, and reinforcement learning to achieve more reliable and interpretable agricultural input optimization.

## 3. Materials and Methods

### 3.1. Data Collection

**Figure 1:**
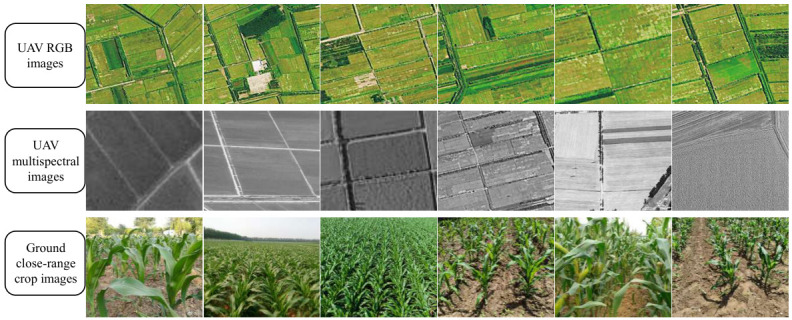
Samples of visual dataset, including UAV RGB images, UAV multispectral images and Ground close-range crop images.

The data used in this study were collected from the Smart Agriculture Demonstration Base in the Hetao Irrigation District, Wuyuan County, Bayannur City, Inner Mongolia Autonomous Region, China, together with supplementary online data sources. The collection period extended from April 2024 to October 2024, covering the main production stages of the crop, including early growth, vegetative accumulation, flowering and fruiting, pest and disease control, and mature harvesting, as shown in Table 1 and Figure 1. This region is characterized by typical irrigated agriculture, and certain differences exist among plots in terms of soil water content, irrigation conditions, management intensity, and crop growth status. Therefore, it provides a suitable scenario for modeling the relationships among agricultural inputs, outputs, and economic returns. Visual data were mainly obtained through unmanned aerial vehicle cruising and ground fixed-camera acquisition. The unmanned aerial vehicle platform was equipped with RGB and multispectral cameras, and low-altitude imaging was conducted over the experimental plots along predefined flight routes during key crop growth stages. Image acquisition was mainly arranged in the morning under sunny or slightly cloudy weather conditions. Ground fixed cameras were installed at the edges of representative plots, and close-range crop images were regularly acquired from fixed viewing angles. Environmental data were continuously collected by field automatic weather stations, including air temperature, relative humidity, rainfall, light intensity, wind speed, and solar radiation, with the sampling period covering the entire production cycle. Soil data were obtained from soil sensors buried near the root zone, mainly including soil moisture, soil temperature, electrical conductivity, and pH. Agricultural management data were derived from the farm management platform, manual record sheets, and operation equipment logs, including fertilization date, fertilizer type, fertilization amount, irrigation time, irrigation amount, pesticide type, spraying intensity, labor input, and machinery operation cost. After harvesting, yield data were obtained through plot-level weighing records, cost data were calculated based on water, fertilizer, pesticide, labor, energy, and machinery operation records, and profit data were computed by integrating yield, quality grade, and contemporaneous market prices. The multimodal dataset consists of synchronized crop images, meteorological observations, soil measurements, and agricultural management records collected throughout the growing season. All modalities were aligned according to predefined agricultural decision windows, within which observations from different sources were temporally synchronized and aggregated into a unified multimodal sample. Image data underwent quality inspection and normalization, while sensor measurements were cleaned to remove abnormal values and missing observations were completed using temporal interpolation when appropriate. Agricultural management records and corresponding economic outcomes were manually verified using field management logs to ensure annotation consistency. Finally, all features were normalized before multimodal state construction.

### 3.2. Data Augmentation

Multi-source agricultural data require unified processing before being fed into the model. Since meteorological data, soil sensor data, agricultural management records, and crop image data are obtained from different devices and recording systems, their sampling frequencies, data scales, and noise sources are inconsistent. Meteorological stations and soil sensors usually generate continuous time series, but they are susceptible to equipment offline events, communication interruptions, and extreme weather conditions. Image data can reflect crop phenotypic status, but they are affected by illumination, occlusion, shooting angle, and cluttered backgrounds. Although agricultural management records can describe key input behaviors such as fertilization, irrigation, and pesticide application, uneven recording intervals and incomplete manual entries are frequently observed. Therefore, standardization, missing-value imputation, image augmentation, and time-window alignment were performed on different modalities before model training, so that stable input representations could be formed at a unified plot–time-window scale. This processing procedure was not only used for data cleaning, but also designed to ensure that subsequent cross-modal spatiotemporal alignment, causal profit modeling, and reinforcement learning decision-making could be conducted under consistent temporal semantics.

For meteorological and soil time series, continuous observations were divided into several fixed-length local temporal segments. This operation was adopted because crop growth responses to environmental changes are usually cumulative and delayed, and temperature, humidity, or soil moisture at a single time point cannot sufficiently describe the growth conditions of a specific stage. Let the multidimensional sensor variable collected from a plot at time *t* be denoted as $x_{t}\in R^{d}$, where *d* represents the variable dimension, including temperature, humidity, rainfall, light intensity, soil moisture, soil electrical conductivity, and other variables. Given the window length *L* and sliding step *s*, the *i*-th time window can be expressed as:

(1) $$X_{i}=\left[x_{is},x_{is+1},\cdots ,x_{is+L-1}\right]\in R^{L\times d}.$$

Through sliding-window processing, the original continuous sequence was converted into a set of agricultural growth segments with fixed lengths, each corresponding to environmental and soil variation processes within a certain temporal range. Because different variables have distinct physical meanings and numerical ranges, such as temperature measured in degrees Celsius, rainfall measured in millimeters, and soil electrical conductivity measured in conductivity units, direct model input may cause variables with larger numerical scales to dominate the training process. To reduce the influence of dimensional differences, each variable in the training set was standardized according to its mean and standard deviation:

(2) $${\tilde{x}}_{t,j}=\frac{x_{t,j}-\mu_{j}}{\sigma_{j}+\epsilon }.$$

Here, $x_{t,j}$ denotes the original observation of the *j*-th variable at time *t*, $\mu_{j}$ and $\sigma_{j}$ denote the mean and standard deviation of this variable in the training set, respectively, and ϵ is a small constant used to prevent division by zero. The standardized variables are more suitable for gradient-based optimization and allow the model to focus on temporal variation trends rather than being dominated by the original numerical ranges.

Missing sensor data are commonly encountered in practical field acquisition. Short-term missing values are usually caused by communication interruption or temporary device offline events. Since such missing values generally do not change the overall variation trend of the variable, temporal interpolation was used for preliminary imputation. If the variable $x_{t,j}$ is missing at time *t*, and $t_{a}$ and $t_{b}$ denote the nearest valid observation times before and after the missing point, respectively, the imputed value can be estimated as:

(3) $${\hat{x}}_{t,j}=x_{t_{a},j}+\frac{t-t_{a}}{t_{b}-t_{a}}\left(x_{t_{b},j}-x_{t_{a},j}\right).$$

This method can maintain temporal continuity in meteorological and soil variables and is suitable for variables with relatively smooth changes, such as temperature, humidity, and soil moisture. However, when rainfall, strong illumination, irrigation, or sudden meteorological events occur, environmental variables may exhibit obvious nonlinear fluctuations, and simple linear interpolation may be insufficient for recovering the true variation pattern. For long-term missing values or simultaneous missing values in multiple variables, a Transformer-based imputation model was further adopted. Let the time series containing missing positions be denoted as X, and let the missing indicator matrix be denoted as M, where $M_{t,j}=1$ indicates that an observation exists at a certain position and $M_{t,j}=0$ indicates that the position is missing. After the masked sequence is fed into the Transformer, the contextual hidden representation can be obtained as:

(4) $$H=\mathrm{Transformer}\left(X⊙M+E_{pos}\right).$$

where ⊙ denotes element-wise multiplication, $E_{pos}$ denotes the temporal positional encoding, and H denotes the hidden representation learned from contextual relationships. Subsequently, the values at missing positions are estimated by a mapping function $f_{\theta }\left(\cdot \right)$:

(5) $$\hat{X}=f_{\theta }\left(H\right).$$

To generate reasonable values at missing positions while preserving consistency with existing observations, a mask-based reconstruction loss was used during training:

(6) $$L_{imp}=\frac{1}{|\Omega_{m}|}\sum_{\left(t,j\right)\in \Omega_{m}}{\left({\hat{x}}_{t,j}-x_{t,j}\right)}^{2}.$$

where $\Omega_{m}$ denotes the set of training positions used for simulated or real missing-value reconstruction, ${\hat{x}}_{t,j}$ is the predicted value, and $x_{t,j}$ is the true observation. In this manner, missing data imputation does not rely solely on adjacent time points, but also exploits historical states, inter-variable relationships, and temporal contextual information.

The processing of image data was mainly intended to improve the adaptability of the model to complex field environments. Crop images are often affected by illumination intensity, shadow occlusion, leaf overlap, rain and fog, shooting height, and cluttered backgrounds. If training samples cover only limited shooting conditions, superficial features associated with specific illumination or background conditions may be learned, leading to performance degradation in cross-plot or cross-year applications. To alleviate this problem, random cropping, color perturbation, and occlusion simulation were applied to images without changing crop category, growth status, or disease semantics. Let the original image be denoted as I, and the augmentation process can be expressed as a random transformation function:

(7) $$\tilde{I}=T\left(I;\alpha \right).$$

where α denotes random augmentation parameters, including cropping ratio, brightness variation amplitude, contrast perturbation intensity, and occlusion region size. Random cropping was used to simulate local crop regions acquired by unmanned aerial vehicles and ground cameras under different shooting distances, angles, and fields of view. This process can be written as:

(8) $$\tilde{I}=\mathrm{Resize}\left(\mathrm{Crop}(I,r_{x},r_{y},r_{w},r_{h})\right).$$

where $r_{x}$ and $r_{y}$ denote the coordinates of the upper-left corner of the cropped region, and $r_{w}$ and $r_{h}$ denote the width and height of the cropped region. Color perturbation was used to simulate color distribution variations caused by different illumination conditions, weather conditions, and acquisition times. Let $I_{rgb}$ denote the three-channel pixel values of the image; the color augmentation can be written as:

(9) $${\tilde{I}}_{rgb}=\beta_{c}I_{rgb}+\beta_{b}.$$

where $\beta_{c}$ denotes the contrast perturbation coefficient and $\beta_{b}$ denotes the brightness offset. For shadows, leaf occlusion, or local incomplete imaging, random occlusion masks were used to construct more complex visual disturbances. Let O denote the occlusion mask, and let $I_{bg}$ denote the filled background or random noise. The occlusion augmentation can be expressed as:

(10) $$\tilde{I}=I⊙\left(1-O\right)+I_{bg}⊙O.$$

where positions with a value of 1 in O indicate occluded regions, and positions with a value of 0 indicate retained original image regions. After these augmentation operations, the model can be exposed to more diverse visual variations during training, thereby reducing its dependence on a single illumination condition, background, or shooting angle.

Since the task of this study focuses on the relationship between agricultural inputs and profits, temporal correspondence among different modalities is particularly important. Meteorological and soil sensors are usually sampled at minute-level or hourly-level frequencies, agricultural management records are mostly stored by day or operation batch, and unmanned aerial vehicle and ground images are often acquired at intervals of several days or one week. If these data are not uniformly aligned in time, crop images, environmental variables, and input records within the same growth stage may not be correctly matched, thereby affecting multimodal fusion and causal modeling. Therefore, plots and time windows were used as the basic indices, and different modal data were mapped to a common time set $T*=t_{1}^{*},t_{2}^{*},\cdots ,t_{N}^{*}$. Let the original sampling time set of modality *m* be denoted as $T^{m}$. Its reconstructed feature at the unified time t* is defined as:

(11) $$z_{t*}^{m}=\sum_{t\in T^{m}}w\left(t,t*\right)z_{t}^{m}.$$

where $z_{t}^{m}$ denotes the feature representation of modality *m* at the original time *t*, and w(t,t*) denotes the temporal distance weight. Observations closer to the unified time point usually better reflect the state of the current time window; therefore, the weight function is defined as:

(12) $$w\left(t,t*\right)=\frac{\exp\left(-\frac{|t-t*|}{\tau }\right)}{\sum_{t^{\prime }\in T^{m}}\exp\left(-\frac{|t^{\prime }-t*|}{\tau }\right)}.$$

where τ is the temporal decay coefficient, which controls the influence range of neighboring time points on the current window. This method enables low-frequency image and management record information to be preserved while forming comparable temporal correspondence with high-frequency sensor data.

After standardization, imputation, augmentation, and temporal alignment, data from different modalities were combined into unified samples. For the data of the *i*-th plot within the *k*-th time window, the multimodal input representation is formulated as:

(13) $$S_{i,k}=\left\{{\tilde{X}}_{i,k}^{e},{\tilde{X}}_{i,k}^{s},{\tilde{I}}_{i,k}^{v},{\tilde{A}}_{i,k}^{m}\right\}.$$

where ${\tilde{X}}_{i,k}^{e}$ denotes the preprocessed meteorological sequence, ${\tilde{X}}_{i,k}^{s}$ denotes the preprocessed soil sequence, ${\tilde{I}}_{i,k}^{v}$ denotes the augmented visual image, and ${\tilde{A}}_{i,k}^{m}$ denotes the aligned agricultural management input records. Through this unified representation, environmental status, soil background, crop visual phenotype, and historical input behavior can be simultaneously obtained by the model within the same time window, thereby providing consistent data inputs for subsequent cross-modal spatiotemporal alignment, input–output causal modeling, and economic return reinforcement learning decision-making in the MAION framework.

### 3.3. Proposed Method

#### 3.3.1. Overall

The proposed MAION model is designed to establish a unified modeling pipeline from multimodal agricultural state perception to economic profit-oriented input decision-making. The preprocessed visual data, environmental time-series data, soil data, and agricultural management data are first fed into their corresponding modality encoders to obtain initial latent representations from different information sources. The visual branch is mainly used to extract phenotypic information such as crop canopy structure, growth status, pest and disease risk, and maturity level. The environmental branch performs temporal encoding for continuously changing variables, including temperature, humidity, light intensity, rainfall, and soil status. The management operation branch transforms fertilization, irrigation, pesticide application, and other agricultural inputs into learnable behavioral features. Since these features differ in sampling frequency, spatial scale, and semantic hierarchy, direct concatenation may cause information mismatch and may be insufficient to support subsequent profit prediction and strategy optimization. Therefore, a cross-modal spatiotemporal alignment module is further introduced to map visual phenotypes, environmental changes, soil states, and management operations into a shared agricultural state space under the same plot–time-window scale. Through temporal encoding and cross-modal attention mechanisms, correspondence among different data sources can be established. Crop visual features can be associated with key environmental variables during the same period, environmental sequences can be used to explain changes in crop status, and management operations can be matched with the crop state and external conditions under which they occur. The aligned comprehensive agricultural state representation is then fed into the input–output causal profit modeling module. In this module, yield, cost, and profit are not treated as mutually independent ordinary regression targets. Instead, input behaviors such as fertilization, irrigation, and pesticide application are regarded as intervenable variables, and yield changes, cost changes, and profit changes under different input conditions are estimated according to the current agricultural state. In this way, the intrinsic relationships among agricultural inputs, production outcomes, and economic returns can be characterized. Yield prediction, cost prediction, and net profit prediction are simultaneously generated, thereby providing economic feedback for subsequent strategy optimization. Finally, the profit-driven reinforcement learning decision module takes the aligned agricultural state and causal profit estimates as environmental feedback, treats fertilization amount, irrigation amount, and pesticide application intensity as optimizable actions, and incorporates long-term net profit, input cost, resource consumption, and risk penalty into the reward function. The policy network generates the input scheme for the next stage according to the current crop status, environmental conditions, and historical input information, and the strategy is continuously refined through value evaluation. As a result, an input decision policy oriented toward long-term net profit maximization can be learned. Through the above pipeline, multimodal feature encoding, spatiotemporal semantic alignment, causal profit estimation, and dynamic input decision-making are connected into a continuous modeling process. Accordingly, agricultural intelligent systems can be extended from a single prediction task to precision input, cost control, and profit optimization tasks.

#### 3.3.2. Cross-Modal Spatiotemporal Alignment Module

The cross-modal spatiotemporal alignment module is used to integrate visual phenotypes, environmental sequences, soil states, and management operations within a unified agricultural decision window, thereby alleviating inconsistencies among different modalities in sampling frequency, temporal granularity, and semantic expression.

**Figure 2:**
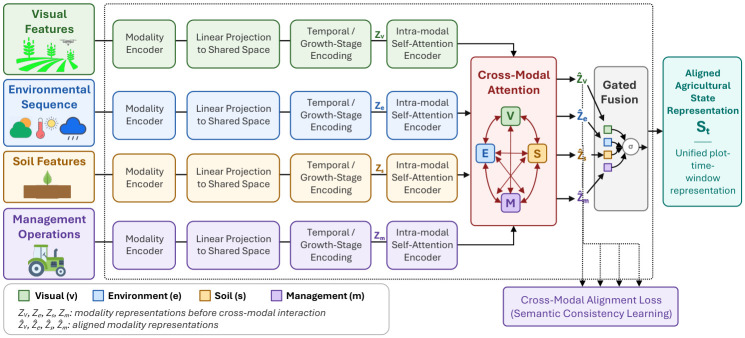
The cross-modal spatiotemporal alignment module projects visual, environmental, soil, and management features into a shared space and integrates them through temporal encoding, intra-modal self-attention, cross-modal attention, gated fusion, and alignment loss to obtain a unified plot–time-window agricultural state representation.

As shown in Figure 2, let the visual features, environmental features, soil features, and management operation features output by the front-end encoders be denoted as $Z_{v}\in R^{T_{v}\times d_{v}}$, $Z_{e}\in R^{T_{e}\times d_{e}}$, $Z_{s}\in R^{T_{s}\times d_{s}}$, and $Z_{m}\in R^{T_{m}\times d_{m}}$, respectively, where $T_{v}$, $T_{e}$, $T_{s}$, and $T_{m}$ denote the temporal lengths of different modalities, and $d_{v}$, $d_{e}$, $d_{s}$, and $d_{m}$ denote the corresponding original feature dimensions. It should be noted that the novelty of the proposed cross-modal spatiotemporal alignment module does not lie in the temporal decay weighting or the cross-modal attention operators themselves, both of which are widely adopted in existing multimodal learning studies. Instead, the distinctive contribution lies in constructing a unified plot–time-window agricultural state representation for profit-oriented decision making. Rather than performing feature-level fusion or simple timestamp synchronization, the proposed framework projects heterogeneous observations, including crop images, environmental measurements, soil conditions, and management records with substantially different sampling frequencies and semantic granularities, into a common agricultural decision window. Consequently, temporal weighting is used to reconstruct asynchronous observations into consistent decision states, while cross-modal attention establishes semantic correspondence among crop phenotypes, environmental dynamics, soil conditions, and historical management behaviors. The resulting aligned representation provides a unified state space that directly supports the subsequent causal profit modeling and reinforcement learning modules, thereby connecting multimodal perception with agricultural economic decision optimization. Since the temporal scales and feature dimensions of different modalities are not unified, learnable linear mappings are first used to project them into a shared space with dimension *d*:

(14) $${\bar{Z}}_{k}=Z_{k}W_{k}+b_{k},\;k\in v,e,s,m.$$

where $W_{k}$ and $b_{k}$ denote the projection matrix and bias term of the *k*-th modality, respectively. To preserve temporal position information and growth-stage information in agricultural production, temporal positional encoding and growth-stage encoding are added to the projected modality representations:

(15) $${\tilde{Z}}_{k}={\bar{Z}}_{k}+P_{k}+G_{k}.$$

where $P_{k}$ denotes the sampling temporal positional encoding, and $G_{k}$ denotes the crop growth-stage or management-cycle encoding. This design prevents the model from fusing different modalities solely according to numerical similarity while ignoring the possibility that the same environmental variable may have different agricultural meanings at different growth stages.

After mapping into the shared space, each modality is first processed by an intra-modal temporal self-attention encoder to capture dynamic relationships within a single modality:

(16) $$Q_{k}={\tilde{Z}}_{k}W_{Q}^{k},\;K_{k}={\tilde{Z}}_{k}W_{K}^{k},\;V_{k}={\tilde{Z}}_{k}W_{V}^{k}.$$

(17) $$H_{k}=\mathrm{MHA}\left(Q_{k},K_{k},V_{k}\right),$$

where MHA(·) denotes the multi-head attention structure, and the number of attention heads, hidden width, feed-forward network dimension, and number of encoding layers are all set as symbolic hyperparameters. This structure models the temporal trend of visual phenotypes, the continuous fluctuation of environmental variables, the gradual evolution of soil states, and the cumulative effects of management operations. On this basis, a cross-modal attention mechanism is further used for information interaction among different modalities. Taking the alignment of visual features with environmental information as an example, the query, key, and value can be expressed as:

(18) $$Q_{v}=H_{v}W_{Q}^{v},\;K_{e}=H_{e}W_{K}^{e},\;V_{e}=H_{e}W_{V}^{e}.$$

The corresponding cross-modal attention weight and environment-enhanced visual representation are defined as:

(19) $$A_{v,e}=\mathrm{softmax}\left(\frac{Q_{v}K_{e}^{⊤}}{\sqrt{d}}\right),\;H_{v\leftarrow e}=A_{v,e}V_{e}.$$

Similarly, bidirectional interactions among visual, environmental, soil, and management modalities are performed through this mechanism. In this way, relationships between crop phenotypic changes and environmental conditions, relationships between input operations and soil-meteorological backgrounds, and consistency between current crop status and historical management behavior can be learned. To reduce the interference of noisy modalities on the fused representation, the final state representation is generated through a gated fusion mechanism:

(20) $$S_{t}=\sum_{k\in v,e,s,m}\alpha_{k}^{t}H_{k}^{t},$$

where

(21) $$\alpha_{k}^{t}=\mathrm{softmax}\left(w^{⊤}\left[H_{k}^{t};H_{cross}^{t}\right]\right).$$

Here, $\alpha_{k}^{t}$ denotes the importance weight of the *k*-th modality in the *t*-th time window, and $H_{cross}^{t}$ denotes the contextual representation after cross-modal interaction. The weight can dynamically vary with plot, growth stage, and data quality; therefore, the influence of unreliable modalities can be reduced when images are missing, sensors are abnormal, or management records are sparse. To enhance semantic consistency among different modalities within the same time window, a cross-modal alignment loss is introduced during training:

(22) $$L_{align}=-\log\frac{\exp(\mathrm{sim}\left(h_{i}^{a},h_{i}^{b}\right)/\tau )}{\sum_{j}\exp\left(\mathrm{sim}\left(h_{i}^{a},h_{j}^{b}\right)/\tau \right)}.$$

This loss pulls the cross-modal representations from the same plot and the same time window closer, while separating representations from different plots or different time windows, thereby improving the discriminability and consistency of the shared latent space. The final output $S_{t}$ is no longer a simple concatenation of multimodal features, but a unified agricultural state representation that integrates crop visual phenotype, environmental driving factors, soil background conditions, and management input context. This module can alleviate temporal mismatch caused by asynchronous acquisition of multi-source agricultural data, enabling subsequent causal profit modeling to estimate the effects of inputs on yield, cost, and profit based on a more reliable state representation. Meanwhile, dynamic, complete, and robust state inputs are also provided for reinforcement learning decision-making.

#### 3.3.3. Profit-Driven Reinforcement Learning Decision Module

The profit-driven reinforcement learning decision module is used to transform causal profit estimation results into executable agricultural input strategies. The input of this module is not the original multimodal data, but the unified agricultural state representation output by the cross-modal spatiotemporal alignment module and the latent economic feedback generated by the causal profit module. It should be noted that the reinforcement learning stage does not interact directly with the real agricultural production environment, nor is it implemented as conventional offline reinforcement learning using fixed historical transition tuples. Instead, the historical multimodal agricultural observations are first used to train the Input–Output Causal Profit Modeling Module, which learns the mapping from agricultural states and management interventions to the corresponding yield, production cost, and economic profit. After convergence, the learned causal model serves as a surrogate agricultural environment. During reinforcement learning training, the policy network continuously interacts with this learned environment, where each agricultural intervention is evaluated by the causal model to generate the corresponding economic reward and updated state representation. This interaction forms a complete state–action–reward–next-state loop for optimizing the policy network while avoiding costly and risky interactions with real agricultural production systems. To improve the practical applicability of the proposed reinforcement learning framework, the agricultural environment is represented by the unified agricultural state generated from the cross-modal spatiotemporal alignment module, together with the economic feedback predicted by the Input–Output Causal Profit Modeling Module and historical management actions. Rather than interacting with real agricultural production systems, the reinforcement learning agent is trained entirely within the learned surrogate agricultural environment, where each intervention is evaluated through the causal profit model to produce the corresponding reward and updated state. The reward jointly considers predicted profit improvement, production cost, resource consumption, and agricultural risk, encouraging economically sustainable decision-making instead of yield maximization alone. Furthermore, all continuous actions are constrained within agronomically feasible ranges, and a behavior regularization term keeps the learned policy close to historically observed management practices. These constraints effectively prevent unrealistic or unsafe agricultural recommendations while improving the reliability and deployability of the proposed decision-making strategy.

**Figure 3:**
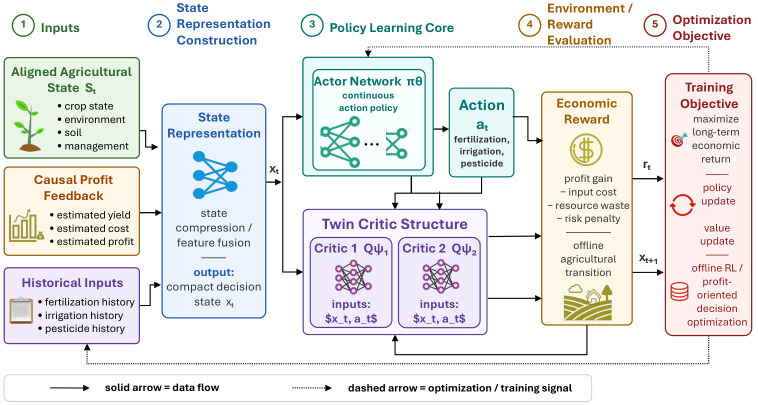
The profit-driven reinforcement learning module integrates aligned agricultural states, causal profit feedback, and historical input records to construct a compact decision state, generate fertilization, irrigation, and pesticide actions through an actor network, and optimize long-term economic returns using twin critics and an economic reward function.

As shown in Figure 3, let the aligned feature map in the *t*-th decision window be denoted as $F_{t}\in R^{H_{t}\times W_{t}\times C_{t}}$, where $H_{t}$, $W_{t}$, and $C_{t}$ denote the height, width, and number of channels of the state feature map, respectively. Meanwhile, the causal profit embedding is denoted as $E_{t}\in R^{d_{e}}$. The spatial state is first compressed into a decision vector through global pooling and linear mapping, and then concatenated with the historical input embedding $M_{t}$ and the economic feedback embedding $E_{t}$ to form the reinforcement learning state representation:

(23) $$q_{t}^{0}=\left[\mathrm{Pool}\left(F_{t}\right)W_{f};E_{t};M_{t}\right].$$

The policy network adopts a multilayer perceptron structure consisting of an input layer, several hidden layers, and an action output layer. The reinforcement learning state is constructed by integrating the aligned agricultural state representation, the causal profit embedding, and historical management actions. Specifically, the predicted yield, production cost, and net profit are first encoded into a compact economic representation before being concatenated with the aligned agricultural state, allowing the policy network to jointly exploit environmental conditions and expected economic outcomes during sequential decision making, as shown in Algorithm 1.
**Algorithm 1** Profit-aware Reinforcement Learning State Construction**Require:** Aligned multimodal sequence $S={\left\{S_{t}\right\}}_{t=1}^{T}$, historical management actions $M={\left\{M_{t}\right\}}_{t=1}^{T}$, intervention sequence $A={\left\{A_{t}\right\}}_{t=1}^{T}$**Ensure:** Profit-aware RL state sequence $X={\left\{x_{t}\right\}}_{t=1}^{T}$  1:**for** t=1,…,T **do**  2:       $z_{t}^{s}\leftarrow f_{s}\left(S_{t}\right)$  3:       $\left({\hat{Y}}_{t},{\hat{C}}_{t},{\hat{R}}_{t}\right)\leftarrow g_{\mathrm{causal}}\left(S_{t},A_{t}\right)$  4:       $z_{t}^{e}\leftarrow f_{e}\left({\hat{Y}}_{t},{\hat{C}}_{t},{\hat{R}}_{t}\right)$  5:       $z_{t}^{m}\leftarrow f_{m}\left(M_{t}\right)$  6:       $x_{t}\leftarrow \left[z_{t}^{s};z_{t}^{e};z_{t}^{m}\right]$  7:       $a_{t}\leftarrow \pi_{\theta }\left(x_{t}\right)$  8:       $\left(r_{t},x_{t+1}\right)\leftarrow E\left(a_{t}\right)$  9:       Update critic network10:       Update actor network11:**end for**12:**return** X

The width of the *l*-th layer is denoted as $h_{l}^{\pi }$, and its forward propagation process is given by:

(24) $$q_{t}^{l}=\mathrm{LN}\left(\sigma (q_{t}^{l-1}W_{l}^{\pi }+b_{l}^{\pi })\right).$$

The output action is constrained within the agriculturally executable range through a bounded transformation:

(25) $$a_{t}=a_{\min}+\frac{a_{\max}-a_{\min}}{2}\left(\tanh(q_{t}^{L_{\pi }}W_{o}^{\pi }+b_{o}^{\pi })+1\right).$$

where $a_{t}$ denotes the fertilization, irrigation, and pesticide input intensity in the current window, and $a_{\min}$ and $a_{\max}$ denote the lower and upper bounds of agricultural input actions, respectively. This constraint can prevent the model from generating excessive input schemes that do not satisfy production safety or resource conditions.

The value network is constructed in parallel with the policy network and is used to estimate the long-term economic value of an input scheme under the current agricultural state. Its input is the state–action combination, and the width of the *l*-th layer is denoted as $h_{l}^{Q}$:

(26) $$v_{t}^{0}=\left[s_{t};a_{t}\right],$$

(27) $$v_{t}^{l}=\sigma \left(v_{t}^{l-1}W_{l}^{Q}+b_{l}^{Q}\right),$$

(28) $$V_{\psi }\left(s_{t},a_{t}\right)=v_{t}^{L_{Q}}W_{o}^{Q}+b_{o}^{Q}.$$

The reward function no longer considers only yield increase. Instead, marginal income, input cost, resource consumption, and agricultural risk are jointly incorporated:

(29) $$\rho_{t}=\omega_{y}\Delta {\hat{Y}}_{t}+\omega_{r}\Delta {\hat{R}}_{t}-\omega_{c}{\hat{C}}_{t}-\omega_{u}{\left|a_{t}\right|}_{2}^{2}-\omega_{z}\Omega_{t},$$

where $\Delta {\hat{Y}}_{t}$ and $\Delta {\hat{R}}_{t}$ denote the predicted yield gain and profit gain brought by the current action, respectively, ${\hat{C}}_{t}$ denotes the input cost, and $\Omega_{t}$ denotes the risk penalty caused by insufficient or excessive input. The policy is updated according to an economic value maximization objective, and training stability is improved through a value residual:

(30) $$\delta_{t}=\rho_{t}+\gamma V_{\bar{\psi }}\left(s_{t+1},\pi_{\bar{\theta }}\left(s_{t+1}\right)\right)-V_{\psi }\left(s_{t},a_{t}\right),$$

(31) $$L_{Q}=E\left[\delta_{t}^{2}\right],\;L_{\pi }=-E\left[V_{\psi }\left(s_{t},\pi_{\theta }\left(s_{t}\right)\right)\right]+\lambda E[|\pi_{\theta }\left(s_{t}\right)-a_{t}^{obs}{|}_{2}^{2}].$$

The constraint term in $L_{\pi }$ is used to limit excessive deviation of the policy from historically feasible agricultural operations, making it more suitable for offline agricultural data scenarios. Its stability can be explained from the perspective of contraction mapping. Let the economic value update operator be denoted as T. For any two value functions $U_{1}$ and $U_{2}$, the following inequality holds:

(32) $$|TU_{1}-TU_{2}{|}_{\infty }\leq \gamma {\left|U_{1}-U_{2}\right|}_{\infty }.$$

Since 0<γ<1, T is a contraction mapping. Therefore, a unique fixed point exists, and the value estimate can gradually approach a stable economic return function during iteration. The advantage of this module lies in its ability to generate continuous, dynamic, and cost-constrained input strategies under the joint influence of crop status, environmental pressure, and historical inputs, thereby shifting agricultural decision-making from yield orientation to net-profit orientation. For the task considered in this study, this module helps reduce ineffective water, fertilizer, and pesticide inputs, improve the input–output ratio, and maintain more robust economic decision-making under extreme weather, pest and disease risk, or resource price fluctuations.

#### 3.3.4. Input–Output Causal Profit Modeling Module

The input–output causal profit modeling module is used to characterize the potential effects of different input behaviors on yield, cost, and net profit based on the unified agricultural state representation. The proposed causal profit modeling module is formulated under the Potential Outcomes framework. Irrigation, fertilization, and pesticide application are treated as intervention variables, while crop yield, production cost, and economic profit are regarded as outcome variables. The aligned multimodal agricultural state representation, including crop phenotypes, meteorological conditions, soil properties, and historical management records, serves as the observed confounders that jointly influence both treatment assignment and agricultural outcomes. To reduce treatment-selection bias, inverse probability weighting (IPW) is combined with latent representation balancing to improve the distributional consistency among treatment groups before estimating counterfactual outcomes. The proposed framework operates under the commonly adopted assumptions of consistency, positivity, and conditional ignorability, under which intervention effects can be estimated from observational agricultural data. It should be emphasized that the objective of this module is to estimate reliable intervention effects under these assumptions rather than to achieve universal causal identification under arbitrary unobserved confounding.

**Figure 4:**
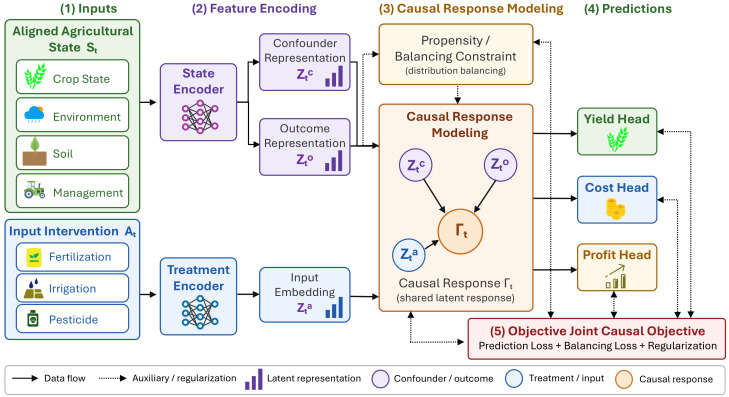
The causal input–output profit modeling module encodes aligned agricultural states and input interventions into confounder, outcome, and treatment representations, and estimates yield, cost, and profit through causal response modeling, balancing constraints, and multi-task prediction heads.

As shown in Figure 4, let the agricultural state feature output by the cross-modal spatiotemporal alignment module be denoted as $S_{t}\in R^{H_{s}\times W_{s}\times C_{s}}$, where $H_{s}$, $W_{s}$, and $C_{s}$ denote the height, width, and number of channels of the state feature map, respectively. The proposed causal profit modeling module is formulated under the Potential Outcomes framework (Rubin Causal Model). Fertilization, irrigation, and pesticide application are treated as interventions, while yield, production cost, and net profit are regarded as potential outcomes. The aligned multimodal agricultural state representation serves as observed covariates that capture the major environmental, soil, crop, and management factors. Under the commonly adopted conditional ignorability assumption, the proposed model estimates intervention effects using inverse probability weighting (IPW) together with latent distribution balancing to mitigate treatment-selection bias in observational agricultural data. It should be noted that the objective of this module is not to achieve full causal identification under arbitrary unobserved confounding, but rather to provide more reliable intervention effect estimation for profit-oriented agricultural decision-making using real-world multimodal observational data. A state compression branch is first constructed, in which $S_{t}$ is fed into a state representation network composed of $L_{s}$ convolutional units. The convolution kernel size of the *l*-th layer is $k_{l}^{s}\times k_{l}^{s}$, the input channel is $C_{l-1}^{s}$, the output channel is $C_{l}^{s}$, the stride is $r_{l}^{s}$, and the padding is $p_{l}^{s}$. The spatial size change is given by:

(33) $$H_{l}^{s}=\left\lfloor \frac{H_{l-1}^{s}+2p_{l}^{s}-k_{l}^{s}}{r_{l}^{s}}\right\rfloor +1,\;W_{l}^{s}=\left\lfloor \frac{W_{l-1}^{s}+2p_{l}^{s}-k_{l}^{s}}{r_{l}^{s}}\right\rfloor +1.$$

After convolution, normalization, activation, and global aggregation, the state embedding $z_{t}^{s}$ can be obtained. Input variables such as fertilization, irrigation, and pesticide application are represented as $A_{t}\in R^{d_{a}}$ and fed into an input encoder composed of $L_{a}$ fully connected layers. The width of the *l*-th layer is $d_{l}^{a}$, and the input embedding $z_{t}^{a}$ is obtained. To reduce dependence on superficial correlations between inputs and outcomes, a confounder disentanglement branch is further introduced, in which the agricultural state is divided into the confounder representation $z_{t}^{c}$ related to input selection and the outcome representation $z_{t}^{o}$ related to crop response:

(34) $$z_{t}^{c}=f_{c}\left(z_{t}^{s};\Theta_{c}\right),\;z_{t}^{o}=f_{o}\left(z_{t}^{s};\Theta_{o}\right),\;z_{t}^{a}=f_{a}\left(A_{t};\Theta_{a}\right).$$

A dual-path profit estimation structure is then adopted. One path is used to learn yield, cost, and profit under factual observation conditions, while the other path is used to estimate counterfactual results after input changes. For any candidate input ${\tilde{A}}_{t}$, its counterfactual response representation is defined as:

(35) $$\Gamma_{t}\left({\tilde{A}}_{t}\right)=\Phi \left(\left[z_{t}^{o},f_{a}\left({\tilde{A}}_{t};\Theta_{a}\right),z_{t}^{c}\right];\Theta_{\Gamma }\right).$$

where Φ(·) is composed of $L_{\Gamma }$ multilayer perceptron layers, and the width of the *l*-th layer is $d_{l}^{\Gamma }$. Based on this representation, the yield head, cost head, and profit head are constructed with $L_{y}$, $L_{c}$, and $L_{r}$ fully connected layers, respectively, and the preceding causal response representation is shared to ensure a consistent input response basis among the three economic variables:

(36) $${\hat{Y}}_{t}\left({\tilde{A}}_{t}\right)=g_{y}\left(\Gamma_{t}\left({\tilde{A}}_{t}\right)\right),\;{\hat{C}}_{t}\left({\tilde{A}}_{t}\right)=g_{c}\left(\Gamma_{t}\left({\tilde{A}}_{t}\right)\right),\;{\hat{R}}_{t}\left({\tilde{A}}_{t}\right)=g_{r}\left(\left[\Gamma_{t}\left({\tilde{A}}_{t}\right),{\hat{Y}}_{t}\left({\tilde{A}}_{t}\right),{\hat{C}}_{t}\left({\tilde{A}}_{t}\right)\right]\right).$$

To enhance comparability among different input levels, a propensity score constraint is introduced during training. The historical probability of input occurrence is estimated using $z_{t}^{c}$, and inverse probability weighting is used to correct the bias of the observed distribution:

(37) $$\omega_{t}=\frac{1}{p(A_{t}|z_{t}^{c})+\epsilon },\;L_{obs}=E\left[\omega_{t}\left(\ell_{y}+\ell_{c}+\ell_{r}\right)\right].$$

Meanwhile, a distribution-distance-based causal regularization term is used to constrain different input groups to remain balanced in the latent space:

(38) $$L_{dis}=\underset{{\left|f\right|}_{H}\leq 1}{\sup}\left|E_{A_{t}∼P_{1}}f\left(z_{t}^{o}\right)-E_{A_{t}∼P_{2}}f\left(z_{t}^{o}\right)\right|.$$

The final optimization objective is expressed as:

(39) $$L_{CY}=L_{obs}+\lambda_{d}L_{dis}+\lambda_{m}{\left|\frac{\partial {\hat{R}}_{t}}{\partial A_{t}}\right|}_{2}^{2}+\lambda_{p}{\left|\Theta \right|}_{2}^{2}.$$

Mathematically, when the representation distribution distance among different input groups is compressed, the counterfactual estimation error can be jointly controlled by the factual error and the distribution discrepancy. For any candidate input *a*, there exists a constant η such that:

(40) $$\varepsilon_{cf}\left(a\right)\leq \varepsilon_{f}\left(a\right)+\eta L_{dis},$$

where $\varepsilon_{cfotesthemulti-headattentionstructure,andthenumberofatt}\left(a\right)$ denotes the counterfactual profit estimation error, and $\varepsilon_{f}\left(a\right)$ denotes the factual observation error. This inequality indicates that when the observed prediction error is reduced and the latent distribution discrepancy among input groups is narrowed, the profit estimation for unexecuted input schemes becomes more reliable. When applied to the task in this study, this module can estimate the marginal effects of increasing or decreasing water, fertilizer, and pesticide inputs on yield, cost, and profit under given crop status, environmental conditions, and historical management background, thereby providing more reliable economic feedback for subsequent reinforcement learning decisions. Compared with ordinary regression models, this design can reduce confounding bias caused by climate, soil, and farmer experience. Thus, not only the current profit level can be estimated, but the potential profit changes after input adjustment can also be analyzed, making the module more suitable for input optimization, cost control, and net profit improvement in smart agriculture.

## 4. Results and Discussion

### 4.1. Experimental Configuration

#### 4.1.1. Hardware and Software Platform

All experiments were conducted on a high-performance deep learning workstation. The computing platform was equipped with a multi-core Intel Xeon processor (Intel, Santa Clara, CA, USA), an NVIDIA RTX 4090 GPU (Nvidia, Santa Clara, CA, USA), 128 GB of memory, and a 2 TB NVMe solid-state drive. During the experiments, the GPU was mainly used for computationally intensive tasks, including visual feature extraction, Transformer-based temporal modeling, multimodal network training, and reinforcement learning policy updates. The CPU was mainly responsible for data loading, sample partitioning, preprocessing, traditional machine learning model training, and cross-validation scheduling. Since the dataset used in this study contains crop images, meteorological and soil time series, agricultural management records, and economic labels, this hardware configuration was able to support joint modeling of multi-source agricultural data and ensure training efficiency and result stability across baseline comparisons, ablation experiments, and complete MAION model evaluations. The software environment was built on the Ubuntu operating system, and Python 3.10 was used as the primary programming language. Deep learning models were implemented using PyTorch 2.12. Random Forest, LSTM, GRU, Transformer, and related neural network modules were trained and tested under a unified experimental framework, while traditional machine learning methods were mainly implemented using Scikit-learn and XGBoost. Data cleaning, statistical analysis, and sample organization were performed using NumPy, Pandas, and SciPy, respectively. Image preprocessing and augmentation were implemented with OpenCV and Torchvision, and experimental visualization and metric statistics were conducted using Matplotlib 3.11.1. To reduce the influence of random initialization, data partitioning, and batch ordering on experimental results, random seeds were fixed in all experiments. Training epochs, learning rate changes, loss curves, model parameters, and final evaluation metrics were uniformly recorded, so that comparisons among different models could be kept as consistent as possible. To evaluate the robustness of the proposed framework, all experiments were repeated five times using different random seeds, and the results are reported as the mean ± standard deviation. Furthermore, statistical significance was assessed by comparing MAION with the strongest baseline using paired statistical tests, where differences with p<0.05 were considered statistically significant. These analyses provide a more rigorous evaluation of the stability and reliability of the proposed framework.

To ensure a fair evaluation and prevent potential data leakage, the dataset was partitioned at the agricultural plot level rather than at the sample level. All temporal windows originating from the same plot were assigned exclusively to either the training, validation, or testing subset, preventing observations from the same field from appearing in multiple partitions. Moreover, chronological ordering was strictly preserved during sample construction, ensuring that only historical observations were used to predict subsequent agricultural decisions. Consequently, neither spatial leakage between agricultural plots nor temporal leakage across decision windows occurred during model development and evaluation. For data partitioning, the dataset was split into training, validation, and test sets at a ratio of 70%:15%:15%. The training set was used for parameter learning, the validation set was used for hyperparameter selection, model selection, and early stopping, and the test set was used only for final performance evaluation. The sliding window length of the time-series data was set to L=14, and the sliding step was set to s=1, so that continuous variations in meteorological, soil, and management variables over short periods could be retained. The image input size was uniformly resized to 224×224, the batch size was set to 32, and the maximum number of training epochs was set to 200. AdamW was adopted as the optimizer, with an initial learning rate of $1\times 10^{-4}$, a weight decay coefficient of $1\times 10^{-5}$, and a Dropout ratio of 0.2. When the validation loss did not decrease for 20 consecutive epochs, early stopping was triggered to reduce the risk of overfitting. Model structural parameters were kept consistent to ensure comparability among different experiments. The hidden dimension of the multimodal encoder was set to 256, the number of Transformer layers was set to 4, the number of attention heads was set to 8, and the feed-forward network dimension was set to 512. In the reinforcement learning decision module, the discount factor γ was set to 0.99, the soft update coefficient τ was set to 0.005, the replay buffer size was set to 100,000, and the learning rates of both the policy network and value network were set to $3\times 10^{-4}$. In addition, 5-fold cross-validation was adopted to further examine the stability of the model results. The training data were divided into 5 non-overlapping subsets, one subset was selected as the validation subset each time, and the remaining subsets were used for model training. The mean results of the 5 experimental runs were finally reported. This setting reduces the randomness caused by a single data split and makes the experimental conclusions more robust.

#### 4.1.2. Baseline Models and Evaluation Metrics

To comprehensively evaluate the effectiveness of the MAION framework, Random Forest [58], XGBoost [59], LSTM [60], GRU [61], Transformer [62], Multimodal Transformer [63], Late Fusion Net [64], DDPG [65], PPO [66], and SAC [67] were selected as comparison methods. Random Forest is a representative ensemble learning method that reduces the fluctuation of a single model through averaging or voting over multiple decision trees, and it is suitable for processing structured agricultural features at a certain scale. XGBoost is based on gradient-boosted decision trees and progressively corrects the residual errors of the previous round of models, which usually provides strong fitting ability for structured data prediction tasks. LSTM and GRU are both recurrent neural networks. LSTM preserves long-term dependencies through input, forget, and output gates, while GRU models sequential variations with a more compact gating structure. Therefore, both models are suitable for agricultural time-series modeling involving meteorological, soil, and management variables. Transformer establishes direct relationships among different time steps through the self-attention mechanism and can identify key environmental changes over longer crop growth periods. Multimodal Transformer is used to enhance information interaction among images, sensors, and management records. Late Fusion Net first extracts modality-specific features independently and then performs fusion at the decision layer; its structure is simpler and can serve as a multimodal fusion baseline. DDPG, PPO, and SAC were used for comparison in input strategy optimization. DDPG is applicable to refined input control in continuous action spaces, PPO improves training stability by restricting policy update magnitudes, and SAC maintains a balance between profit optimization and policy exploration based on the maximum entropy principle, making it more suitable for long-term decision-making tasks in complex dynamic environments.

The models were evaluated from three perspectives: prediction accuracy, economic benefit, and policy performance. For yield, cost, and profit prediction tasks, RMSE, MAE, and $R^{2}$ were used to measure the error and goodness of fit between predicted and observed values. For agricultural economic benefit evaluation, Net Profit Improvement, Input–Output Ratio, and Cost Efficiency Gain were used to measure the net profit increase, input–output relationship, and cost efficiency improvement compared with baseline methods, respectively. For input decision policy evaluation, Cumulative Reward, Policy Stability, and Regret Analysis were used to reflect cumulative returns, action smoothness, and the gap from the ideal policy over the whole decision cycle. The metrics are defined as follows:

(41) $$RMSE=\sqrt{\frac{1}{N}\sum_{i=1}^{N}{\left(y_{i}-{\hat{y}}_{i}\right)}^{2}}.$$

(42) $$MAE=\frac{1}{N}\sum_{i=1}^{N}\left|y_{i}-{\hat{y}}_{i}\right|.$$

(43) $$R^{2}=1-\frac{\sum_{i=1}^{N}{\left(y_{i}-{\hat{y}}_{i}\right)}^{2}}{\sum_{i=1}^{N}{\left(y_{i}-\bar{y}\right)}^{2}}.$$

(44) $$NPI=\frac{R_{m}-R_{b}}{R_{b}}\times 100\%.$$

(45) $$IOR=\frac{R}{C}.$$

(46) $$CEG=\frac{CE_{m}-CE_{b}}{CE_{b}}\times 100\%.$$

(47) $$CR=\sum_{t=1}^{T}\gamma^{t-1}r_{t}.$$

(48) $$PS=\frac{1}{T-1}\sum_{t=2}^{T}{\left|\pi_{t}-\pi_{t-1}\right|}_{2}^{2}.$$

(49) $$Regret=\sum_{t=1}^{T}\left(r_{t}^{*}-r_{t}\right).$$

Here, *N* denotes the number of samples, $y_{i}$ and ${\hat{y}}_{i}$ denote the observed and predicted values of the *i*-th sample, respectively, and $\bar{y}$ denotes the mean of the observed values. $R_{m}$ and $R_{b}$ denote the net profits obtained by the proposed method and the baseline method, respectively, *R* denotes the total return, *C* denotes the total input cost, and ${CE}_{m}$ and ${CE}_{b}$ denote the cost efficiencies of the proposed method and the baseline method, respectively. *T* denotes the length of the decision cycle, γ denotes the discount factor, $r_{t}$ denotes the reward at step *t*, $\pi_{t}$ denotes the policy at step *t*, and $r_{t}^{*}$ denotes the reward corresponding to the ideal policy at step *t*. Through these metrics, prediction accuracy, economic benefit improvement, and input policy quality can be evaluated simultaneously, thereby providing a more comprehensive reflection of the application value of the model in smart agricultural input optimization scenarios.

### 4.2. Prediction Performance Comparison

The purpose of this experiment was to compare the fundamental modeling capabilities of different types of models in yield, cost, and net profit prediction tasks, and to verify whether MAION can maintain a stable advantage in multi-objective agricultural economic prediction. In this experiment, not only yield prediction but also cost and profit prediction were considered, because agricultural input optimization cannot rely solely on yield estimation. If only yield can be predicted while input costs and net profits cannot be accurately estimated, the subsequently generated input strategy may still tend toward a high-input, high-output, but low-profit scheme.

**Figure 5:**
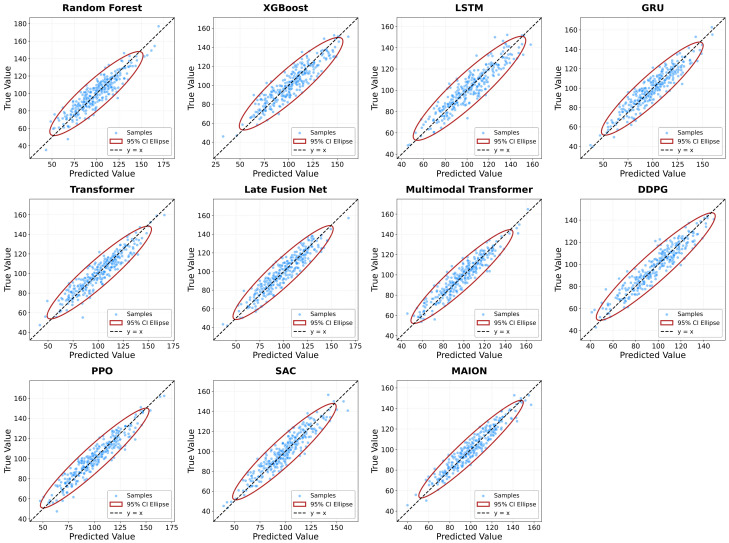
Prediction scatter matrix of different models with 95% confidence ellipses and the y=x reference line, showing that MAION produces predictions more closely aligned with the true values.

As shown in Table 2 and Figure 5, Random Forest and XGBoost exhibit competitive baseline performance among traditional machine learning methods across the three prediction tasks. XGBoost performs better than Random Forest overall, indicating that the gradient boosting mechanism has stronger fitting ability for structured agricultural variables. Compared with traditional models, LSTM and GRU further reduce RMSE and MAE, suggesting that historical dependencies in meteorological, soil, and management sequences play an important role in prediction. Transformer achieves further improvements across all three tasks, indicating that the self-attention mechanism is more capable than recurrent structures of capturing key environmental changes over long time spans. Late Fusion Net and Multimodal Transformer outperform single time-series models, demonstrating the complementarity among visual, environmental, and management data. Although DDPG, PPO, and SAC are decision-oriented models, certain performance is also obtained after adaptation to prediction-based evaluation, with SAC achieving the best result among them. MAION obtains the best performance in yield, cost, and profit prediction, especially in net profit prediction, where RMSE is reduced to 0.646 and $R^{2}$ reaches 0.883, indicating that agricultural economic outcomes are more sufficiently characterized by the proposed model.

The relatively high yield-prediction $R^{2}$ of 0.914 should be interpreted in the context of the present experimental setting rather than as a universally attainable level of performance. Although plot-level partitioning and chronological ordering were adopted to reduce direct information leakage between the training and test sets, the data were collected mainly from a single smart-agriculture demonstration base during one growing season. Within this setting, crop varieties, irrigation systems, management practices, and cultivation schedules were relatively consistent across the experimental plots, resulting in lower spatial and management heterogeneity than would be expected in a large-scale multi-region or multi-crop dataset. In addition, the data-collection period did not include severe extreme-weather events or widespread pest and disease outbreaks, which reduced uncontrolled variability in yield formation. MAION also jointly utilizes crop visual phenotypes, meteorological observations, soil measurements, and historical management records, providing more comprehensive explanatory information than single-source models. Therefore, the reported $R^{2}$ reflects the model performance under a relatively homogeneous irrigated-agriculture scenario, and the limited spatial, temporal, and crop-management variability of the current dataset may have contributed to this value. Further validation using multi-region, multi-crop, and multi-year datasets with greater environmental heterogeneity is required to determine whether a comparable level of performance can be maintained in broader agricultural scenarios.

From a theoretical perspective, the differences among the results of different models are directly related to their mathematical modeling characteristics. Random Forest reduces variance by averaging multiple trees, but each tree still essentially relies on local partitioning, making it difficult to represent long-range temporal dependencies in continuous agricultural processes. XGBoost improves fitting accuracy by progressively correcting residual errors, but its input usually depends on manually organized structured features, and its capacity to represent image phenotypes and cross-modal relationships is limited. LSTM and GRU retain historical states through gating mechanisms and are suitable for processing environmental and soil sequences, but the retention of long-distance dependencies may still be affected by state compression in recurrent structures. Transformer establishes relationships among arbitrary time steps through global self-attention, and therefore has advantages under complex climate fluctuations and growth-stage variations. Late Fusion Net can integrate multimodal information, but the fusion occurs at a relatively late stage, and fine-grained spatiotemporal correspondences among modalities are not fully learned. Multimodal Transformer improves information interaction, but without plot–time-window-oriented alignment constraints, it may still be affected by inconsistent sampling frequencies. DDPG, PPO, and SAC are more suitable for strategy optimization, and prediction is not their core advantage. The advantage of MAION mainly arises from three aspects: cross-modal spatiotemporal alignment reduces modality mismatch, causal profit modeling weakens spurious correlations between input variables and environmental confounders, and the profit-oriented structure enables the model to learn the joint response relationships among inputs, outputs, and economic outcomes rather than merely fitting superficial regression relationships for cost and profit prediction.

### 4.3. Economic Benefit and Decision Performance Comparison

The purpose of this experiment was to evaluate the performance of different models in agricultural input optimization and economic decision-making tasks, with emphasis on net profit improvement, input–output ratio, cost efficiency, cumulative reward, policy stability, and regret. Compared with Table 2, this experiment is closer to the actual production objective, because the ultimate concern of agricultural managers is whether input strategies can lead to higher profits rather than whether prediction errors are lower.

**Figure 6:**
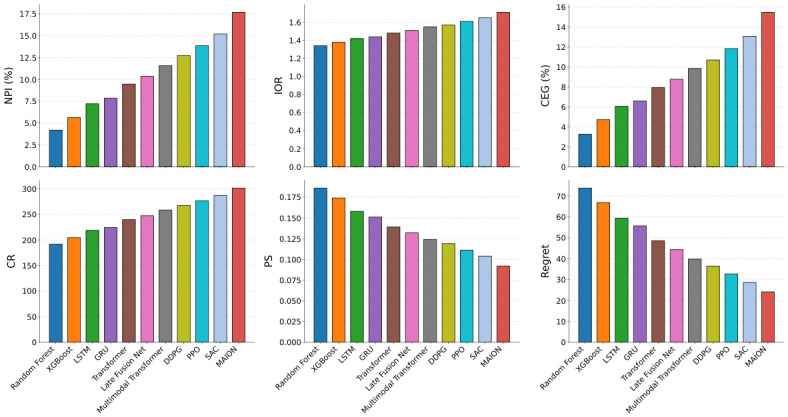
Bar-chart comparison of economic benefit and decision performance across different models, indicating that MAION achieves the highest NPI, IOR, CEG, and CR while maintaining the lowest PS and Regret.

As shown in Table 3 and Figure 6, Random Forest and XGBoost achieve certain economic profit improvements, but their NPI and CR remain relatively low. This indicates that traditional prediction models can provide yield or profit estimates, but it is difficult for them to form long-term optimal continuous input strategies. LSTM and GRU achieve improved economic indicators, suggesting that time-series information is helpful for input judgment. Transformer, Late Fusion Net, and Multimodal Transformer further improve NPI, IOR, and CR, indicating that multi-source information fusion enables economic decisions to better reflect actual production states. The economic decision performance of DDPG, PPO, and SAC is significantly better than that of ordinary prediction models. Among them, SAC obtains an NPI of 15.21% and a cumulative reward of 286.92, demonstrating the advantage of reinforcement learning methods in continuous input control. MAION achieves the best performance across all indicators, with an NPI of 17.68%, an IOR of 1.71, and a CEG of 15.46%. Meanwhile, the lowest PS and Regret are also achieved, indicating that its strategy not only yields higher profits but also changes more smoothly and is closer to the ideal strategy.

This result can be explained by the objective functions and policy learning mechanisms of different models. Traditional machine learning and ordinary deep learning models are usually trained to minimize prediction error. Even when prediction results are accurate, optimal input decisions may not be directly generated, because long-term economic consequences of different actions are not explicitly compared by such models. The decision basis of Random Forest and XGBoost mainly comes from static feature mapping, and future state transitions are not characterized. LSTM, GRU, and Transformer can use historical environmental changes, but they still essentially function as prediction models, and their feedback mechanisms for action selection are relatively weak. Late Fusion Net and Multimodal Transformer improve state estimation, but net profit maximization is not incorporated as a core optimization objective. DDPG is suitable for continuous action control, but it is susceptible to value estimation bias. PPO improves training stability by limiting the magnitude of policy updates; therefore, its PS is better than that of DDPG. SAC introduces entropy regularization and maintains a balance between profit optimization and policy exploration, which explains its best performance among the reinforcement learning baselines. MAION further outperforms SAC because its policy learning is not directly based on raw states, but on agricultural states and economic feedback obtained after cross-modal alignment and causal profit modeling. In other words, the policy network receives not only the current plot state, but also the yield, cost, and profit responses that may be caused by different input behaviors. Such a state representation reduces ineffective exploration, improves the reliability of value estimation, and enables the reward function to more accurately penalize resource waste and excessive costs, thereby forming a more robust long-term profit optimization strategy.

### 4.4. Ablation Study

The purpose of this experiment was to verify the contribution of different internal modules and modalities in MAION to the overall performance, and to determine whether the performance improvement originates from the collaborative effect of the complete structure.

**Figure 7:**
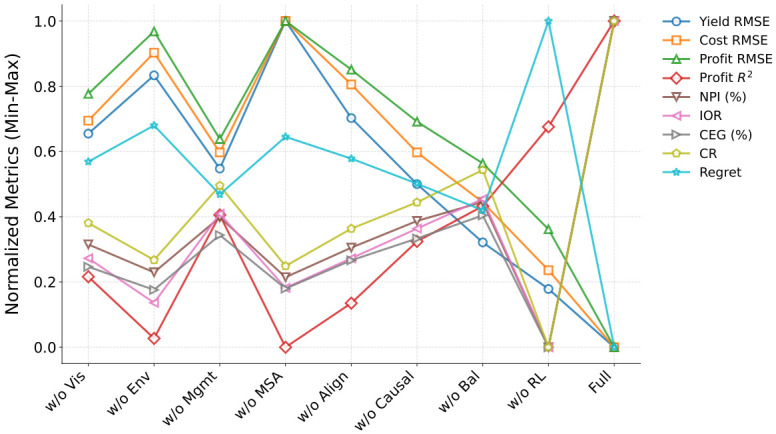
Normalized ablation curves of MAION variants across prediction, economic benefit, and decision metrics, demonstrating the performance degradation caused by removing key modalities or modules.

As shown in Table 4 and Figure 7, removing any modality or key module leads to performance degradation. After the visual modality is removed, Yield RMSE, Profit RMSE, and NPI all deteriorate significantly, indicating that crop phenotypic information is important for yield and profit assessment. The degradation caused by removing the environmental modality is more pronounced, with Yield RMSE increasing to 0.657 and NPI decreasing to 11.47%, indicating that meteorological and soil environments are fundamental factors affecting agricultural input returns. After management records are removed, prediction can still be performed using visual and environmental information, but economic indicators decline, showing that historical fertilization, irrigation, and pesticide inputs are irreplaceable for profit estimation and strategy learning. After the MSA module is removed, one of the most obvious overall performance declines is observed, suggesting that simple fusion cannot effectively handle the asynchronicity of multimodal agricultural data. After Alignment Loss is removed, the attention-based fusion structure is still retained, but its performance is inferior to that of the complete model, indicating that explicit alignment constraints help improve the consistency of the shared space. After the Causal Yield Module and Balancing Constraint are removed, both profit prediction and decision indicators decline, showing that causal modeling and distribution balancing are effective in reducing confounding bias. After the Economic RL Module is removed, prediction errors change relatively little, but NPI, IOR, CEG, CR, and Regret deteriorate substantially, indicating that the main contribution of the reinforcement learning module lies in strategy optimization rather than pure prediction accuracy improvement.

From a theoretical perspective, the ablation results reflect the complementary roles of perception, causal estimation, and decision optimization in MAION. The visual modality provides the actual phenotypic state of crops and reflects the integrated effects of environment and management behaviors on crops. Environmental and soil modalities provide external driving factors and determine the response boundaries of crops to water, fertilizer, and pesticide inputs. Management records provide historical trajectories of intervenable actions, allowing the model to understand the dynamic relationships between input behaviors and outcomes. The function of the MSA module is to map data with different frequencies and semantic levels into a unified time window. If this module is removed, the model can only rely on coarse concatenation or weak fusion, and images, environments, and input records from different periods may be incorrectly associated. Alignment Loss improves the aggregation of the latent space by pulling cross-modal representations under the same state closer; therefore, cross-modal representation quality is degraded after its removal. The Causal Yield Module distinguishes state factors, input factors, and potential confounders, so that superficial correlations in historical samples are not merely learned. The Balancing Constraint further reduces the representation distribution discrepancy among different input groups, which is beneficial for counterfactual profit estimation. The Economic RL Module transforms profit estimation into continuous input strategies. If it is removed, yield and profit can still be predicted, but the mechanism for adjusting input actions according to long-term objectives is lost. The best performance of the complete MAION indicates that its advantage does not arise from a single module alone, but from the continuous transmission among multimodal state alignment, causal profit estimation, and economic reinforcement learning.

### 4.5. Robustness Evaluation Under Diverse Experimental Conditions

The purpose of this experiment was to examine the stability of the model under cross-scenario transfer and missing-modality conditions. Agricultural data are characterized by obvious plot differences, inter-annual climate differences, and equipment-missing problems. Therefore, good performance in a fixed training scenario alone is insufficient to demonstrate practical applicability. Cross-plot, cross-year, missing visual modality, missing environmental modality, and missing management records were included in this experiment to simulate realistic uncertainties that may be encountered during model deployment.

**Figure 8:**
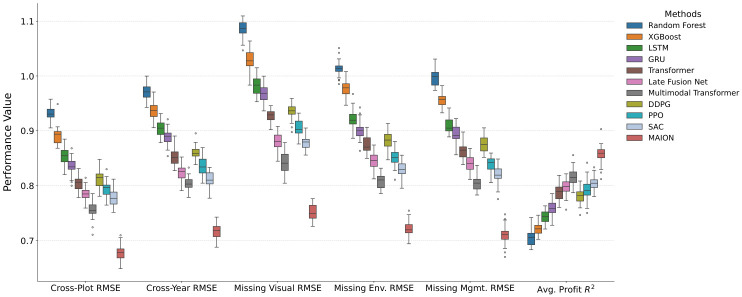
Boxplot comparison of cross-plot generalization, cross-year generalization, and missing-modality robustness, showing that MAION achieves lower error distributions and higher average profit $R^{2}$ than the baseline models.

As shown in Table 5 and Figure 8, Random Forest and XGBoost exhibit relatively high errors under cross-scenario conditions, indicating that traditional structured models are sensitive to distribution shifts. LSTM and GRU outperform traditional models under cross-year and missing-modality conditions, but they remain insufficient for fully handling modality missingness and regional differences. Transformer achieves further improvement owing to its global attention mechanism. Late Fusion Net and Multimodal Transformer are more stable under missing-modality conditions, indicating that multimodal information can buffer the impact of single-source data failure. DDPG, PPO, and SAC perform less prominently in the generalization experiment than in the economic decision experiment, suggesting that reinforcement learning strategies are easily affected by scenario changes when stable state representations are lacking. MAION achieves the lowest RMSE and the highest Avg. Profit $R^{2}$ in all scenarios. Specifically, the cross-plot RMSE is 0.681, the cross-year RMSE is 0.716, and the RMSE under missing management records remains 0.708, indicating stronger adaptability to spatial transfer, temporal transfer, and incomplete information.

This result is closely related to how each model handles distribution shifts and missing information. Random Forest and XGBoost rely on feature partitions and boosted residuals formed from training data. When the feature distribution of a test plot or year changes, existing partitioning rules cannot sufficiently cover new environmental combinations. Although LSTM and GRU can capture temporal dependencies, their hidden states mainly propagate along a single sequence, and explicit compensation mechanisms for cross-plot spatial differences and modality missingness are lacking. Transformer selects key time steps through attention weights and is therefore more robust in cross-year experiments; however, if the missing information belongs to a key modality, reliable information sources may still be insufficient. Since each modality is encoded independently in Late Fusion Net, basic outputs can be more easily maintained than in early fusion when some modalities are missing, but inter-modal interactions remain weak. Multimodal Transformer can learn relationships among modalities, resulting in stronger overall robustness; nevertheless, without alignment loss and gating mechanisms, missing or noisy modalities may still interfere with fusion results. The generalization ability of reinforcement learning methods is strongly affected by changes in the state space, and action preferences formed in the training environment may not be applicable to new plots or new years. MAION establishes a unified state space through cross-modal spatiotemporal alignment, reduces the weights of abnormal or missing modalities through gated fusion, and mitigates the influence of environmental confounders on input–profit estimation through the causal profit module. This structure enables a relatively reliable agricultural state to be reconstructed from the remaining modalities under plot differences, inter-annual climate variations, and partial data missingness, while maintaining stable estimation of net profit.

### 4.6. Computational Efficiency and Deployment Analysis

To further evaluate the practical applicability of the proposed framework, the computational complexity and inference efficiency of MAION were analyzed from both model-level and module-level perspectives. As summarized in Table 6, MAION contains 52.7 million parameters and requires 41.3 GFLOPs, which are higher than those of conventional sequence models and Transformer-based baselines. This increase is expected because MAION integrates visual feature extraction, cross-modal spatiotemporal alignment, causal profit modeling, and reinforcement learning into a unified decision-making framework. Unlike prediction-oriented models that perform only feature extraction and regression, MAION additionally models intervention effects and sequential decision optimization, inevitably introducing additional computational overhead.

Despite its relatively larger model size, MAION maintains an average inference latency of only 21.4 ms per sample, corresponding to an inference throughput of 47 FPS. Such latency satisfies the real-time requirements of typical smart agriculture applications, where agricultural management decisions are usually generated at the level of minutes or hours rather than milliseconds. Therefore, the additional computational cost introduced by the proposed framework remains acceptable considering the substantial improvements in prediction accuracy and economic decision-making performance. To better understand the computational characteristics of different components, Table 7 further reports the inference latency of each functional module. The visual encoder accounts for 48.6% of the total inference time, indicating that feature extraction from high-dimensional image data dominates the computational cost of the entire framework. By contrast, the multimodal alignment module, causal profit modeling module, and reinforcement learning policy network require only 19.6%, 14.5%, and 17.3% of the total latency, respectively. These results indicate that the proposed multimodal alignment and causal reasoning mechanisms introduce only moderate computational overhead compared with visual perception. It is also worth noting that the reinforcement learning policy network is computationally lightweight during deployment. The expensive policy optimization procedure is performed offline during model training, whereas online decision making only requires a single forward inference through the trained actor network. Consequently, the deployed system does not repeatedly execute reinforcement learning optimization, making the online inference considerably more efficient than the offline training process. Overall, the experimental results indicate that the computational overhead introduced by the proposed multimodal alignment, causal modeling, and reinforcement learning modules is moderate relative to the achieved improvements in prediction accuracy and profit-oriented decision-making, demonstrating a favorable trade-off between performance and deployment efficiency.

### 4.7. Discussion

The experimental results indicate that agricultural input optimization should not be understood merely as a yield prediction problem, but should instead be analyzed within specific production management scenarios. In typical irrigated agricultural regions such as the Hetao Irrigation District in Wuyuan County, clear differences exist among plots in soil water content, irrigation accessibility, management intensity, and crop growth status. In practical management, producers frequently need to determine whether additional irrigation is required for a given plot, whether fertilizer input should be reduced, whether pest and disease prevention should be advanced, and whether these operations can eventually generate sufficient incremental profit. Traditional experience-based management usually relies on manual field inspection and historical habits. However, when the plot area is large and sensor data and image data continuously increase, it becomes difficult for manual experience to simultaneously process meteorological conditions, soil status, crop phenotypes, and cost information. The value of MAION lies in organizing these originally scattered information sources into the same decision window, so that input recommendations are no longer based on a single observation, but are determined by jointly considering the current crop status, environmental background, and historical input behavior. Although the experiments demonstrate the effectiveness of MAION under representative irrigated agricultural conditions, the current validation is limited to data collected from the Hetao Irrigation District in Inner Mongolia. Therefore, the present study does not claim comprehensive validation across different geographical regions, crop species, or agricultural production systems. The proposed framework is designed around multimodal agricultural state representation, causal profit modeling, and profit-oriented reinforcement learning rather than crop-specific handcrafted features, suggesting potential transferability to other agricultural scenarios. Nevertheless, different climatic conditions, cultivation practices, and crop characteristics may introduce distinct environmental responses and management strategies. Consequently, further validation using datasets collected from humid southern regions, protected agriculture, orchards, and additional crop types will be an important direction for future work to comprehensively evaluate the cross-region and cross-crop generalization capability of the proposed framework.

From the perspective of practical application, the proposed method is more suitable for production scenarios in which water, fertilizer, and pesticide inputs are frequent and cost-sensitive. For example, during consecutive high-temperature and low-rainfall periods, decisions based solely on soil moisture thresholds may tend to increase irrigation. However, if images show that crop growth remains stable and historical irrigation records indicate that earlier inputs have already been high, a strategy of moderately delaying irrigation or reducing irrigation intensity may be generated by the model to avoid unnecessary water costs. Conversely, when soil moisture has not yet fallen below the threshold but crop canopy color, meteorological evapotranspiration conditions, and historical input records jointly indicate increasing growth pressure, potential yield reduction risks can be identified earlier by the model. A similar situation applies to pesticide application. If visual images and environmental data indicate increasing pest and disease risk, but the model determines that the marginal return from continued high-intensity spraying is limited, the strategy will tend to control spraying intensity rather than simply pursue the lowest possible risk. Such a decision-making process is closer to the trade-off process in real agricultural management, in which a relatively reasonable balance among yield, cost, risk, and profit is sought. The ablation experiment also indicates that different data sources play distinct roles in agricultural economic decision-making. Visual data reflect the current crop performance, environmental and soil data explain the background in which such performance is formed, and management records provide the trajectory of past input behaviors. If any type of information is missing, the model’s judgment of input effects becomes incomplete. In profit prediction and strategy optimization in particular, the role of management records is not limited to providing cost information. More importantly, they help the model understand under what input level a certain yield result was formed. The causal profit modeling module further strengthens this interpretability, enabling the model to distinguish yield changes caused by environmental differences from profit changes caused by input behaviors themselves. Therefore, the outputs of MAION can be used not only for automated input recommendation, but also as a basis for farm managers to review plot management effects. By comparing input and profit differences among plots under similar environmental conditions, redundant links in water, fertilizer, and pesticide use can be identified, and empirical judgment can be gradually transformed into quantifiable and traceable management evidence. The relatively high prediction accuracy achieved by MAION should be interpreted together with the characteristics of the experimental dataset. All experiments were conducted using data collected from the same smart agriculture demonstration base in the Hetao Irrigation District, where crop varieties, irrigation systems, management practices, and cultivation schedules were relatively consistent, resulting in lower environmental heterogeneity than large-scale multi-region agricultural datasets. In addition, MAION jointly exploits crop images, meteorological observations, soil measurements, and agricultural management records, providing a more comprehensive representation of crop growth and management conditions than conventional single-source prediction models. Furthermore, the data collection period did not include severe extreme weather events or widespread pest and disease outbreaks, thereby reducing unpredictable disturbances during crop production. Therefore, the reported $R^{2}$ values primarily reflect the characteristics of the current experimental scenario and should not be interpreted as universally achievable performance across arbitrary agricultural environments. Future work will further evaluate the proposed framework using multi-region and multi-year datasets with greater environmental variability.

### 4.8. Limitation and Future Work

Although the proposed MAION framework shows favorable performance in yield, cost, net profit prediction, and input strategy optimization, several limitations remain. The data used in this study were mainly obtained from the Hetao Irrigation District in Wuyuan County, Bayannur City, Inner Mongolia Autonomous Region, and related online data sources. Although this region is characterized by typical irrigated agriculture, its climate conditions, crop types, soil structure, and management patterns still exhibit certain regional characteristics. Therefore, the applicability of the model to other crops, rainfed agricultural areas, facility agriculture, or humid environments in southern regions still needs to be further verified. In addition, although visual, meteorological, soil, and management record data were integrated in this study, economic factors such as market prices, agricultural product quality grading, long-term soil health, and policy subsidies were not fully incorporated into the model, which may affect the granularity of net profit estimation in real business scenarios. The reinforcement learning decision module was mainly optimized based on historical data and simulated feedback, which can reduce the risk of direct field trial-and-error. However, the recommended strategies still need to be validated through long-term tracking in larger-scale real production practice. Future research may further expand cross-regional, cross-crop, and cross-year datasets to enhance the transferability of the model across different agricultural ecological zones. Real-time market prices, quality grades, carbon emissions, water resource constraints, and soil sustainability indicators may also be introduced so that the input optimization objective can better reflect both agricultural operation and ecological protection requirements. In addition, edge computing and farm management platforms may be combined to transform model outputs into more intuitive irrigation, fertilization, and pest and disease control recommendations, thereby improving deployability and practical value in real agricultural production.

## 5. Conclusions

A MAION framework is constructed for smart agriculture, input efficiency improvement, and agricultural economic profit optimization, enabling a transition from multi-source agricultural perception to profit-driven input decision-making. To address the difficulty of synchronously utilizing visual images, meteorological environments, soil states, and agricultural management records in practical production, cross-modal spatiotemporal alignment, input–output causal profit modeling, and economic reinforcement learning decision-making are integrated into a unified framework. As a result, the model can not only predict yield variations, but also further evaluate input costs and net profit responses. This design extends agricultural intelligent modeling from single production-indicator prediction to water, fertilizer, and pesticide input optimization and farm profit improvement, which is more consistent with the requirements of precision management and sustainable development in modern agricultural production. Experimental results show that MAION outperforms Random Forest, XGBoost, LSTM, GRU, Transformer, Multimodal Transformer, and reinforcement learning baseline models in yield, cost, and net profit prediction. Since the task mainly involves regression prediction and continuous decision optimization rather than classification detection, classification metrics such as accuracy, precision, and recall were not adopted. Model accuracy was mainly evaluated using RMSE, MAE, and $R^{2}$. The RMSE values achieved by MAION in yield, cost, and profit prediction are 0.587, 0.531, and 0.648, respectively, with corresponding $R^{2}$ values of 0.914, 0.891, and 0.883, indicating that the relationship between agricultural production outcomes and economic profits can be more stably characterized. In the economic decision-making experiment, MAION obtains a Net Profit Improvement of 17.68%, an Input–Output Ratio of 1.71, and a Cumulative Reward of 301.27, while achieving the lowest policy fluctuation and regret. The ablation study further demonstrates that cross-modal alignment, causal profit modeling, and balancing constraints all make important contributions to model performance improvement. Cross-plot, cross-year, and missing-modality experiments also show that MAION maintains better generalization ability and robustness in complex agricultural scenarios. Overall, a feasible modeling strategy is provided for multi-source agricultural data-driven input optimization, cost control, and profit improvement.

## Figures and Tables

**Table 1:** Collection of multi-source agricultural input–output data.

Data Name	Sensor Type or Data Source	Quantity
UAV RGB Images	UAV-mounted RGB Camera	18,640 images
UAV Multispectral Images	UAV-mounted Multispectral Camera	7820 images
Ground Close-range Crop Images	Field Fixed Camera	42,300 images
Meteorological Environmental Data	Field Automatic Weather Station	263,520 records
Soil Moisture Data	Soil Moisture Sensor	186,400 records
Soil Temperature Data	Soil Temperature Sensor	186,400 records
Soil EC and pH Data	Soil Electrical Conductivity and pH Sensor	124,800 records
Fertilization Records	Farm Management Platform and Manual Record Sheets	3260 records
Irrigation Records	Irrigation Control System Logs	4180 records
Pesticide Spraying Records	Spraying Operation Logs	2740 records
Labor and Machinery Cost Records	Labor Ledger and Equipment Operation Records	2150 records
Plot-level Yield Labels	Plot-level Harvest Weighing Records	1280 records
Production Cost Labels	Water, Fertilizer, Pesticide, Labor, and Machinery Cost Accounting Tables	1280 records
Net Profit Labels	Yield, Quality Grade, and Market Price Records	1280 records

**Table 2:** Prediction performance comparison of different baseline models on yield, cost, and profit estimation.

Method	Yield Prediction			Cost Prediction			Profit Prediction		
	RMSE ↓	MAE ↓	$R^{2}$ ↑	RMSE ↓	MAE ↓	$R^{2}$ ↑	RMSE ↓	MAE ↓	$R^{2}$ ↑
Random Forest	0.841±0.015	0.623±0.009	0.785±0.014	0.733±0.011	0.547±0.008	0.765±0.015	0.911±0.018	0.684±0.011	0.740±0.013
XGBoost	0.797±0.013	0.585±0.010	0.807±0.012	0.707±0.014	0.522±0.008	0.788±0.014	0.873±0.017	0.640±0.009	0.767±0.015
LSTM	0.758±0.012	0.553±0.009	0.822±0.014	0.685±0.011	0.504±0.008	0.802±0.015	0.832±0.014	0.611±0.010	0.794±0.016
GRU	0.743±0.012	0.537±0.009	0.837±0.017	0.670±0.010	0.495±0.008	0.813±0.014	0.817±0.016	0.595±0.010	0.807±0.013
Transformer	0.705±0.013	0.511±0.008	0.859±0.016	0.641±0.012	0.467±0.007	0.834±0.018	0.781±0.013	0.567±0.009	0.825±0.015
Late Fusion Net	0.680±0.011	0.497±0.008	0.869±0.019	0.621±0.010	0.453±0.007	0.844±0.014	0.750±0.013	0.545±0.009	0.838±0.016
Multimodal Transformer	0.656±0.010	0.475±0.007	0.884±0.017	0.596±0.011	0.433±0.006	0.858±0.016	0.723±0.015	0.520±0.009	0.853±0.018
DDPG	0.696±0.011	0.513±0.008	0.863±0.011	0.629±0.010	0.460±0.006	0.836±0.014	0.763±0.014	0.556±0.007	0.834±0.015
PPO	0.671±0.011	0.488±0.008	0.877±0.010	0.613±0.012	0.446±0.006	0.850±0.013	0.741±0.010	0.535±0.008	0.843±0.017
SAC	0.662±0.009	0.480±0.007	0.880±0.018	0.605±0.011	0.438±0.008	0.854±0.014	0.733±0.009	0.525±0.009	0.847±0.013
**MAION**	0.585±0.008	0.422±0.005	0.914±0.006	0.530±0.010	0.384±0.005	0.891±0.017	0.646±0.011	0.463±0.008	0.883±0.007

**Table 3:** Economic benefit and decision performance comparison of different baseline models.

Method	NPI (%) ↑	IOR ↑	CEG (%) ↑	CR ↑	PS ↓	Regret ↓
Random Forest	4.18 ± 0.062	1.34 ± 0.024	3.26 ± 0.060	191.42 ± 2.872	0.186 ± 0.003	73.85 ± 1.182
XGBoost	5.63 ± 0.118	1.38 ± 0.020	4.71 ± 0.094	204.36 ± 3.680	0.174 ± 0.003	66.92 ± 1.271
LSTM	7.21 ± 0.108	1.42 ± 0.025	6.04 ± 0.121	218.57 ± 4.153	0.158 ± 0.003	59.43 ± 1.129
GRU	7.86 ± 0.141	1.44 ± 0.028	6.58 ± 0.118	224.19 ± 3.811	0.151 ± 0.003	55.76 ± 0.948
Transformer	9.47 ± 0.180	1.48 ± 0.027	7.93 ± 0.143	239.68 ± 4.554	0.139 ± 0.002	48.62 ± 0.972
Late Fusion Net	10.36 ± 0.166	1.51 ± 0.029	8.76 ± 0.193	247.25 ± 5.192	0.132 ± 0.002	44.37 ± 0.843
Multimodal Transformer	11.58 ± 0.220	1.55 ± 0.029	9.84 ± 0.187	258.43 ± 4.910	0.124 ± 0.002	39.85 ± 0.717
DDPG	12.74 ± 0.242	1.57 ± 0.027	10.69 ± 0.214	267.31 ± 5.346	0.119 ± 0.002	36.42 ± 0.692
PPO	13.86 ± 0.263	1.61 ± 0.031	11.83 ± 0.248	276.54 ± 5.531	0.111 ± 0.002	32.76 ± 0.590
SAC	15.21 ± 0.319	1.65 ± 0.030	13.07 ± 0.261	286.92 ± 5.451	0.104 ± 0.002	28.64 ± 0.544
**MAION**	**17.68 ± 0.336**	**1.71 ± 0.035**	**15.46 ± 0.294**	**301.27 ± 6.025**	**0.092 ± 0.002**	**24.19 ± 0.435**

**Table 4:** Ablation study of the proposed MAION framework.

Variant	Yield RMSE ↓	Cost RMSE ↓	Profit RMSE ↓	Profit $R^{2}$ ↑	NPI (%) ↑	IOR ↑	CEG (%) ↑	CR ↑	Regret ↓
MAION w/o Visual Modality	0.642 ± 0.010	0.581 ± 0.010	0.721 ± 0.013	0.854 ± 0.016	12.16 ± 0.219	1.55 ± 0.027	9.72 ± 0.185	262.45 ± 5.249	39.68 ± 0.794
MAION w/o Environmental Modality	0.657 ± 0.012	0.596 ± 0.011	0.739 ± 0.015	0.847 ± 0.015	11.47 ± 0.206	1.52 ± 0.026	9.18 ± 0.165	255.34 ± 4.597	42.71 ± 0.897
MAION w/o Management Records	0.633 ± 0.011	0.574 ± 0.010	0.708 ± 0.013	0.861 ± 0.018	12.83 ± 0.256	1.58 ± 0.031	10.46 ± 0.209	269.62 ± 5.393	36.95 ± 0.702
MAION w/o MSA Module	0.671 ± 0.014	0.603 ± 0.011	0.742 ± 0.013	0.846 ± 0.017	11.35 ± 0.216	1.53 ± 0.028	9.21 ± 0.175	254.18 ± 5.084	41.76 ± 0.877
MAION w/o Alignment Loss	0.646 ± 0.012	0.589 ± 0.011	0.728 ± 0.015	0.851 ± 0.016	12.08 ± 0.242	1.55 ± 0.030	9.87 ± 0.187	261.36 ± 4.966	39.94 ± 0.719
MAION w/o Causal Yield Module	0.629 ± 0.011	0.574 ± 0.009	0.713 ± 0.013	0.858 ± 0.017	12.74 ± 0.255	1.57 ± 0.030	10.38 ± 0.197	266.42 ± 5.062	37.85 ± 0.719
MAION w/o Balancing Constraint	0.614 ± 0.010	0.563 ± 0.011	0.701 ± 0.014	0.862 ± 0.019	13.21 ± 0.264	1.59 ± 0.031	10.92 ± 0.218	272.64 ± 5.453	35.62 ± 0.677
MAION w/o Economic RL Module	0.602 ± 0.010	0.548 ± 0.010	0.682 ± 0.012	0.871 ± 0.016	9.62 ± 0.163	1.49 ± 0.027	7.84 ± 0.149	238.57 ± 4.294	51.43 ± 1.029
**Full MAION**	**0.587 ± 0.010**	**0.531 ± 0.009**	**0.648 ± 0.013**	**0.883 ± 0.018**	**17.68 ± 0.353**	**1.71 ± 0.034**	**15.46 ± 0.309**	**301.27 ± 5.724**	**24.19 ± 0.460**

**Table 5:** Cross-scenario generalization and missing-modality robustness comparison.

Method	Cross-Plot RMSE ↓	Cross-Year RMSE ↓	Missing Visual RMSE ↓	Missing Environment RMSE ↓	Missing Management RMSE ↓	Avg. Profit $R^{2}$ ↑
Random Forest	0.934 ± 0.016	0.972 ± 0.019	1.086 ± 0.022	1.014 ± 0.019	0.998 ± 0.018	0.701 ± 0.013
XGBoost	0.891 ± 0.017	0.936 ± 0.018	1.032 ± 0.020	0.976 ± 0.019	0.951 ± 0.016	0.724 ± 0.013
LSTM	0.852 ± 0.016	0.904 ± 0.018	0.984 ± 0.017	0.921 ± 0.018	0.913 ± 0.016	0.748 ± 0.014
GRU	0.837 ± 0.015	0.887 ± 0.014	0.963 ± 0.019	0.904 ± 0.016	0.896 ± 0.017	0.759 ± 0.013
Transformer	0.806 ± 0.014	0.851 ± 0.016	0.927 ± 0.018	0.872 ± 0.017	0.861 ± 0.014	0.781 ± 0.016
Late Fusion Net	0.782 ± 0.013	0.824 ± 0.015	0.881 ± 0.018	0.843 ± 0.014	0.836 ± 0.015	0.799 ± 0.014
Multimodal Transformer	0.754 ± 0.015	0.797 ± 0.013	0.842 ± 0.017	0.811 ± 0.014	0.804 ± 0.015	0.817 ± 0.017
DDPG	0.813 ± 0.016	0.858 ± 0.014	0.936 ± 0.019	0.886 ± 0.017	0.873 ± 0.014	0.776 ± 0.015
PPO	0.791 ± 0.013	0.831 ± 0.017	0.904 ± 0.015	0.854 ± 0.014	0.842 ± 0.016	0.794 ± 0.013
SAC	0.773 ± 0.014	0.809 ± 0.015	0.876 ± 0.017	0.828 ± 0.016	0.816 ± 0.014	0.808 ± 0.016
**MAION**	**0.681 ± 0.012**	**0.716 ± 0.013**	**0.752 ± 0.016**	**0.721 ± 0.013**	**0.708 ± 0.012**	**0.856 ± 0.018**

**Table 6:** Model complexity comparison of different methods in terms of parameter size, computational cost, and inference efficiency.

Model	Params (M)	FLOPs (G)	Inference (ms)	FPS
LSTM	12.8	8.6	6.4	156
GRU	11.5	7.9	5.8	172
Transformer	28.6	22.5	11.2	89
Multimodal Transformer	45.3	35.8	18.5	54
**MAION**	**52.7**	**41.3**	**21.4**	**47**

**Table 7:** Inference latency analysis of individual modules in the proposed MAION framework.

Module	Time (ms)	Percentage
Visual Encoder	10.4	48.6%
Alignment	4.2	19.6%
Causal Module	3.1	14.5%
RL Policy	3.7	17.3%
Total	21.4	100%

## Data Availability

The data presented in this study are available on request from the corresponding author.
